# Dataset of plugging and abandonment status from exploration wells drilled within the Troll gas and oil field in the Norwegian North Sea

**DOI:** 10.1016/j.dib.2021.107165

**Published:** 2021-05-25

**Authors:** Benjamin Emmel, Bastien Dupuy

**Affiliations:** SINTEF Industry, Applied Geoscience Group, P. O. Box 4763 Torgarden, 7465 Trondheim, Norway

**Keywords:** Plugging and Abandonment (P&A), North Sea, Geographic information systems, Well integrity, CO_2_ and hydrogen storage

## Abstract

More than 750 wildcat wells have been drilled in the Norwegian North Sea since 1966. Some of these wells could pose a risk for the environment, climate, and future CO_2_ and hydrogen storage projects by being potential leakage pathways for subsurface gases (mainly CH_4_ and stored CO_2_ and hydrogen). To ensure well integrity, these wells were secured by building cement plugs at crucial positions in the well path before abandoning the well. However, first 2004 the NORSOK d-010 standard defined strict regulation for plugging and abandonment (P&A) of gas and oil wells along the Norwegian continental margin. Here we report data relevant for the quality of a P&A work done on old exploration wells (1979 to 2003) from the Troll gas and oil field in the Norwegian North Sea. The data was extracted from public available well completion reports and the webpage of the Norwegian Petroleum Directorate. The dataset was analysed regarding their availability, plausibility and evaluated towards the present P&A regulations for offshore Norway. Based on twelve single criteria a final P&A score for 31 exploration wells was established, which may be applied to other abandoned wells in the Norwegian North Sea for further analyses. The resulting scores vary from -1 to 23.67 whereby lowest scores indicate wells where monitoring would be recommended. A P&A work evaluation is especially relevant in the Troll area as Norwegian large-scale CO_2_ storage is planned close to this location.

## Specifications Table

**Table:** 

Subject	Geology Ocean and Maritime Engineering Petroleum Engineering Geographical Information System (GIS)
Specific subject area	The goal of plugging and abandonment (P&A) of a gas and oil exploration well is permanent well integrity [1]. However, as indicated by several monitoring studies [2,3], leakage incidents [4], or stated in the Norwegian guideline NORSOK d-010 [5] well barrier materials have limited lifetimes or might not be installed according to present regulated requirements. Therefore, it is desirable to be able to monitor the integrity of P&A'ed wells. Before entering this stage, a good overview of the P&A status and associated well architecture is necessary. This can only be achieved by evaluating old drilling operation reports towards critical parameters such as position of the plugs, cementing of wellbore casing and plugging, and the testing of cement casing/plug integrities.
Type of data	Table Figure GIS shape file
How data were acquired	The data was collected from well completion reports or directly from the Norwegian Petroleum Directorate (NPD) webpage (see below) providing open access data. Additionally, simple calculations for the hole, casing and cement plug volumes were carried out in an excel spreadsheet.
Data format	Raw data Analysed
Parameters for data collection	We investigate exploration wells in the Troll gas and oil field in the Norwegian North Sea. Adjacent to this area CO_2_ storage is anticipated and therefore well integrity is of upper-most importance to prevent unintended CO_2_ migrations through well paths outside the chosen storage unit(s) and structure(s). We focused on exploration wells because these are usually the oldest wells, they are mainly vertical and thus crosscut all major sealing and reservoir rocks, and most data is publicly available.
Description of data collection	Primary data is extracted from the NPD webpage and mined from well completion reports (see below). The primary data contains e.g., date of drilling, date of reporting towards the authorities, well status, hole sizes, casing sizes, vertical depth, plug sizes, plug vertical depths, amount of cement used, applied verification tests. Usually, all information is given in the drilling or operation section, reported in the summary for the different hole/casing intervals, the description of the daily drilling/operation, given in tables and/or shown in schematic drawings. The primary data is then saved in an excel sheet and analysed.
Data source location	Institution: SINTEF Industry City/Town/Region: Trondheim Country: Norway Coordinates of wellbores and links to the primary data sources are given in the *P&A_Troll_DiB.shp* file Primary data sources: Norwegian Petroleum Directorate: - completion reports from all described well bores can be entered via: https://factpages.npd.no/en/wellbore/pageview/exploration/all - wellbore data from shape files were partly used: https://www.npd.no/en/about-us/information-services/open-data/map-services/
Data accessibility	With the article. Instructions for accessing these data: The shape file (*P&A_Troll_DiB.shp* in the folder *Wells*) is given in the supplementary data and can be loaded in a GIS program.

## Value of the Data

• Old gas and oil exploration wells might not be plugged and abandoned following present regulations and thus might pose an environmental risk. The established scoring system can indicate which wells need extra attention including monitoring of the well and plug integrity and assessing potential leakage pathways.
• The dataset can be regarded as a blueprint how to evaluate the plugging and abandonment status of old exploration wells. The dataset is useful for researchers as a dataset and conceptual idea to be further developed. For companies and government institutions it provides information useful during decision making processes without having a detailed engineering and geological knowledge.
• The dataset can be used for definition of monitoring cases of plugged and abandoned wells using e.g., geophysical methods, detailed analysis of wellbores and by authorities for risk assessment (e.g., CO_2_ and hydrogen storage).

## 1 Data Description

We analysed twelve different criteria to conclude with a final score (Table 1). The final score relates to the report quality of P&A activities and can be used for the selection of further detailed document search or for the selection of monitoring targets (e.g., if scores are low). The data can be visualized using geographical information system (GIS) tools such as the QGIS 3.16 [6] the actual version of an open-source GIS software. All data is provided in the shapefile *P&A_Troll_DiB.shp* (supplementary data). In this file, the first 17 fields (giving information about well name, location, depth et.) are copied from the NPD file *wlb.Point.zip* (https://www.npd.no/en/about-us/information-services/open-data/map-services/), fields 17–19 relate to associated image files and fields 19–32 are the new P&A scores, discussed in the following. The field identification name of each score used in the *P&A_Troll_DiB.shp* file is given in brackets.

**Table 1:** Scoring for P&A of exploration wells from the Troll gas and oil filed Norwegian North Sea. *wb_name* gives the well bore name all other scoring criteria are discussed in data description.

wb_name	sc_status	sc_entryYear	sc_plug_da	sc_plug_ab	sc_report	sc_cem_job	sc_cs_ver	sc_plug_jo	sc_plug_len	sc_pl_ver	sc_mil	sc_ind_lk	sc_total
31/2–1	1	0	0	0	2	3.00	1	−6	2.80	0	0	1	**4.80**
31/2–2	1	0	0	0	3	1.80	1	4	2.00	0	0	1	**13.80**
31/2–3	3	0	0	0	3	3.00	1	5	1.80	1	0.5	1	**19.30**
31/2–4	1	0	0	0	2	2.50	2	1	3.00	1	0	1	**13.50**
31/2–5	1	0	0	0	2	1.80	1	3	3.00	1	0.5	1	**14.30**
31/2–6	3	0	0	0	2	2.25	2	3	2.50	2	0.5	1	**18.25**
31/2–7	3	0	0	0	3	3.00	2	6	2.67	2	1	1	**23.67**
31/2–9	3	0	0	0	3	3.00	2	3	3.00	1	0	1	**19.00**
31/2–10	3	0	0	0	3	2.00	2	5	3.00	1	0	1	**20.00**
31/2–11	3	0	0	0	3	2.25	2	3	2.40	2	0.5	1	**19.15**
31/2–12	3	0	0	1	3	3.00	2	4	3.00	1	0.5	1	**21.50**
31/2–13S	3	0	0	0	3	3.00	1	3	3.00	2	0.5	1	**19.50**
31/2–14	3	0	0	0	3	2.25	2	−3	1.00	2	0.5	1	**11.75**
31/2–15	3	0	0	0	3	2.25	2	3	2.20	1	0	1	**17.45**
31/2–16S	3	0	0	0	1	0.00	2	−6	0.00	0	0	1	**1.00**
31/2–17S	3	1	0	0	1	2.00	1	0	0.00	0	0	1	**9.00**
31/2–18	3	1	0	0	2	2.25	1	−6	0.50	0	0	1	**4.75**
31/2–20S	3	1	0	0	0	0.00	1	−6	0.00	0	0	1	**0.00**
31/3–1	3	0	0	0	3	2.40	2	0	2.58	0	0.5	2	**15.48**
31/3–2	3	0	0	0	1	3.00	1	1	1.75	0	0.5	2	**13.25**
31/5–2	1	0	0	0	1	1.80	1	1	2.25	1	0.5	1	**10.55**
31/5–3	3	0	0	0	2	1.80	1	1	3.00	0	0.5	1	**13.30**
31/5–4	3	0	0	0	0	0.00	1	−6	0.00	0	0	1	**−1.00**
31/5–5	1	1	0	0	1	1.50	1	−6	0.00	0	0	1	**0.50**
31/6–1	3	0	0	0	3	2.40	1	1	2.88	1	0.5	2	**16.78**
31/6–2	1	0	0	0	1	3.00	1	6	2.88	1	0.5	2	**18.38**
31/6–4	3	0	0	0	3	1.50	1	4	2.67	1	0	1	**17.17**
31/6–5	3	0	0	0	3	2.40	2	0	2.75	1	0	2	**16.15**
31/6–6	3	0	0	0	3	2.40	2	0	3	1	0.5	2	**16.90**
31/6–7	2	0	0	0	1	0.00	2	3	3	0	0	2	**13.00**
31/6–8	1	0	0	0	1	1.80	1	0	1	0	0	1	**6.80**
**Max value**	**3**	**1**	**1**	**1**	**3**	**3**	**3**	**6**	**3**	**3**	**1**	**2**	**30.00**

### 1.1 Status (*sc_status*)

The NPD gives different status descriptions for exploration wells. An exploration well is regarded as P&A'ed if it is plugged and abandoned and cannot be re-entered for further use. It is important to note that the quality of the P&A job is not evaluated in this status description. A wellbore is plugged if the upper parts of the wellbore can be re-used. Suspension of a well under construction or intervention is defined as a well status, where the well operation is suspended without removing the well control equipment. An exploration well can be temporary abandoned if a blow-out preventer has been removed and well barriers are not monitored. The status junked is regarded for well bores with terminated drilling operations due to technical problems but have been permanently plugged. They are evaluated with the same score as plugged wells. The relationships with reservoir units and potential fluid flow pathways are evaluated in Section 1.8 Plugging job (*sc_plug_jo*)**.** The following status scores are given: P&A: 3; plugged/junked: 2; temporary abandoned/suspended: 1.

### 1.2 Drilling year (*sc_entYear*)

The year of drilling is critical because before 1992 no regulation for P&A were available. In all cases included in this dataset the P&A job was done in the drilling year. In 1992, the Norwegian oil and gas industry introduced the first well barrier schematics for the Norwegian continental shelf. The first full regulation for P&A is given in the NORSOK D-010 standard [5]. All wells drilled before 1992 got a score of 0, wells drilled between 1992 and 2004 got a score of 1 and wells drilled after 2004 got a score of 2.

### 1.3 Plugged date (*sc_plug_da*)

The score is 1 if the finishing date of the plugging operation has been reported by the operator to the NPD, if not the score is 0.

### 1.4 Plugged and abandon date (*sc_plug_ab*)

The score is 1 if the finishing date for the P&A operation has been reported by the operator to the NPD, if not the score is 0.

### 1.5 Reporting (*sc_report*)

Here we evaluate the report quality by mainly checking if the casing and plugging cement jobs are well described and plausibly explained. This includes: a schematic drawing of the P&A job (if available attached in the *P&A_Troll_DiB.shp* file with the field name *image_P&A*), a detailed reporting of the drilling job including description of borehole and casing sizes, reporting of the used amounts of cement for the casing and plugging cement jobs. The report quality can vary from good (3), OK (2), to bad (1). If reporting is not publicly available, the score is 0 and criteria 1.6 to 1.11 will get the lowest scores.

### 1.6 Casing cement job (*sc_cem_job*)

**Fig. 1:**
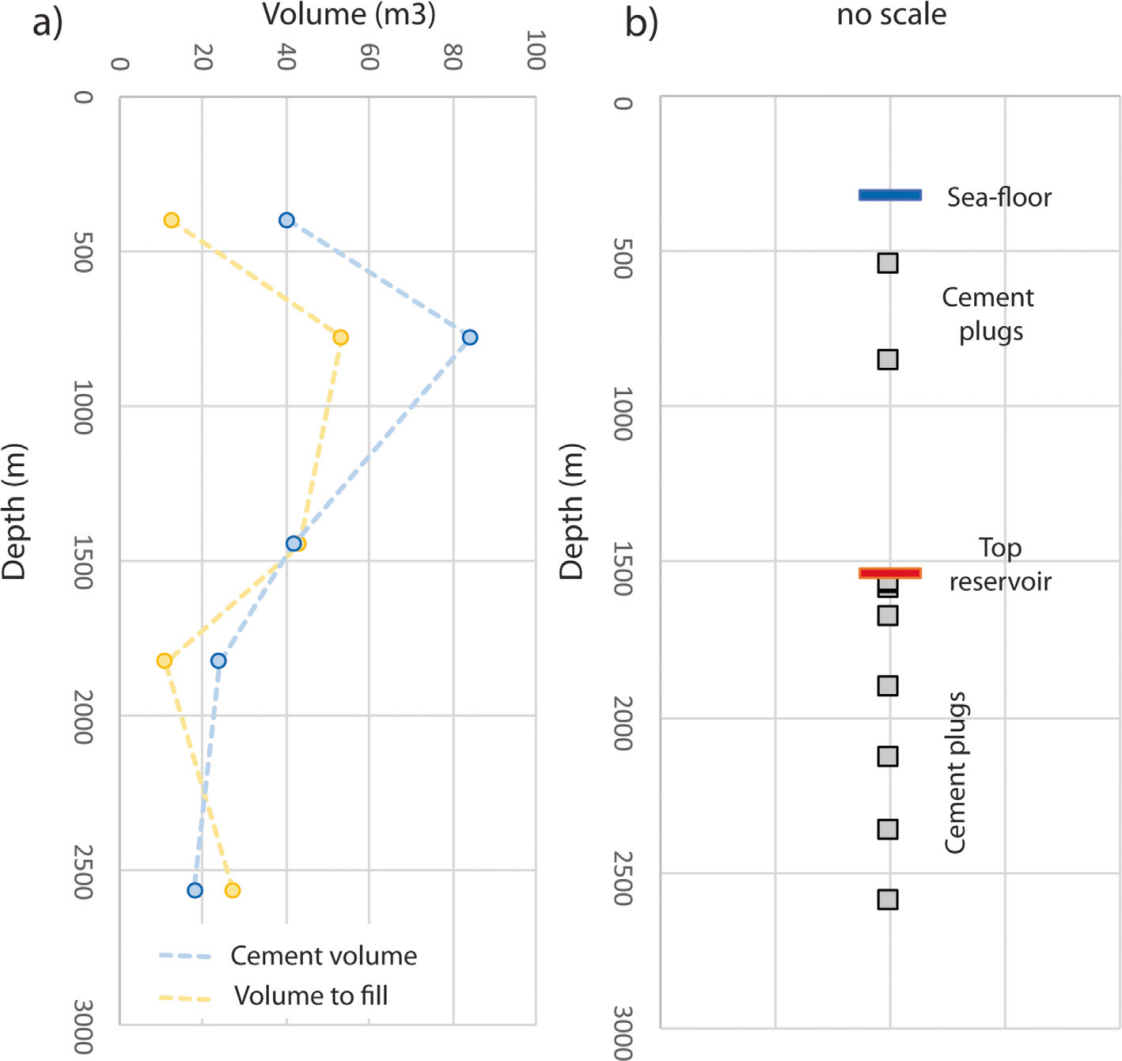
Graphic representation of the casing cement job and the plug position for well 31/2–2. a) The yellow line indicates the volume between the open hole and the casing which is to be filled by cement. The blue line is the volume of cement used between every of the 4 casing intervals. In this case, the cement volumes of the 3rd casing interval and for the open hole (1826 - 2568 m) are less than the hole volume resulting in a cement job score of 3*(3/5) = 1.8 (data is given as in the data repositorium as an excel sheet and figures are in the *P&A_Troll_DiB.shp* file with the field name *cs_cem_P&A*). b) Schematic illustration of plug position in relation to the top of the reservoir and the seafloor (the plug length is not considered in the figure). In this example, abandonment, reservoir, and environmental plugs are in the correct positions. Additionally, we evaluated the anticipated plug length and compare the theoretically needed volumes with the used cement volumes (data is given in the supplementary data file as an excel sheet and figures are attached in the *P&A_Troll_DiB.shp* file under the field name *plugs_P&A*).

In the casing cement job evaluation, we investigate the volume of the drilled hole and the volume of used cement. The NORSOK D10 standard recommends that all zones with flow potential are covered. However, approximately 2/3 of the evaluated abandonment plans (supplementary data *P_&_A_Plans*) aimed for a full cement coverage outside the casing. In general, scores are high (average 2.04) indicating that enough cement was used. For 4 wells cement volumes are not reported and a score of 0 is given. Furthermore, well integrity is identified as one of the major risks for future CO_2_ [7] and hydrogen storage projects [8]. Thus, we decided to evaluate the total volumes of used cement for the full length of the wellbore (including well lead, tail and shoe, without add-ons) but other evaluations strategies might be applicable as well. The volume calculation is done by assuming a cylindric shape of the borehole and the casing. The volume between the open borehole and the casing was calculated by subtracting the casing volume from the open borehole volume. Temperature, pressure and add-ons volumes are neglected in the volume calculations. For the cement we assumed a constant shrinkage factor of 0.66 which agrees with laboratory measurements for chemical shrinkage of normal Portland cement pastes ranging between 0.4 and 0.8 [9]. The total score is calculated by adding the individual scores for every casing interval and normalize it to three. The casing cement job is regarded as sufficient if the cement volume exceeds the volume of the hole. Usually, the amounts of cement are given in sacks (1 sack of Portland cement = 42.64 kg), total amount (kg, t) or as volumes (m^3^). If this information is not provided the amounts are regarded as 0. Fig. 1a gives an example of volume calculations for the casing cement job of well 31/2–2.

### 1.7 Casing cement job verification (*sc_cs_ver*)

For the two uppermost casing intervals the casing cement job is usually verified by visual inspection (camera, divers) of returning cement to surface. In deeper parts of the well, the top of the cement is tagged with the wellhead. Furthermore, leak-off tests give indications of cement/cap rock integrity within and outside the casing. In the deeper parts of the well, logging tools such as cement bounding logging or variable density logs are used to monitor the cement bounding with the casing steel and the cement thicknesses outside of the well casing. The score is 2 if all casing cement jobs are verified. If at least one casing interval has a verified cement job, the score is 1. The score is 0 if verification is not reported.

### 1.8 Plugging job (*sc_plug_jo*)

Permanently plugged and abandoned wells should be protected with an eternal perspective considering the effects of any foreseeable chemical and/or geological processes [5]. We evaluate three plug types, for a more detailed description, we refer to [1]. These are from the deepest to the shallowest wellbore depth:

- Abandonment plug: this well barrier should protect against any potential source of inflow within permeable zones. These plugs fill the deepest parts of the well, sometimes open holes (without a casing).
- Reservoir plug: if a potential source of inflow or a reservoir is exposed e.g., to hydrocarbons a plug is required. Usually, a barrier consists of a primary and secondary well barrier, whereby the secondary well barrier should back-up the primary against a potential source of inflow.
- Environmental plug: this well barrier should isolate the surface/seabed from any potential source of inflow. The environmental isolation plug is installed close to the surface. It is the shallowest well hindrance that isolates open hole annuli from the external environments. In the Troll case, we consider as shallow the upper 500 m of the well or 500 m below seafloor.

Permanent well barriers shall extend across the full cross section of the well, include all annuli and seal both vertically and horizontally [e.g., 10].

In the plugging job evaluation, we consider two criteria. At first, we check if all plugs are available and in the correct position, e.g., is a reservoir plug correctly placed according to the present regulations and regional geological knowledge (e.g., top of a reservoir unit). An example of plug locations versus depth for well 31/2–2 is given in Fig. 1b. Secondly, we compare the theoretical volume to be filled with used cement volumes. For all three plug types we give scores from +2, if the plugs are in place and filled with enough cement, to −2 if the plugs are not installed. This gives a score ranging between −6 and +6 when considering the mandatory and here discussed plugs.

### 1.9 Plug length (*sc_pl_len*)

An important issue regarding plug integrity is the plug length. Currently, requirements for plug length vary between different countries and regulatory regimes [1,10]. For example, in the Norwegian North Sea the required plug length is 100 m (50 m if a mechanical bridge plug is used as a foundation), compared to a required plug length of 30 m (100 ft) at the UK side. The scores vary between 3 (≥ 100 m), 2 (50 - 100 m), 1 (30–50 m) and 0 (0–30 m).

### 1.10 Plug cement job verification (*sc_pl_ver*)

The vertical position of a plug can be verified by tagging. The stability of a plug can be weight tested using e.g., the weight on bit and/or pressure tested. The score ranges between 2 (all tested) and 0 (no tests).

### 1.11 Milling, reaming or perforation (*sc_mil*)

Sometimes the casing is removed (milled, reamed) to ensure cement integrity with the surrounding rocks through the full cross-section of the well [10]. By removing the casing steel, the plug quality does not depend on the cement bounding with the inside and outside casing steel. Through perforation of a well, a reservoir gets connected through the well bore and the benefit of perforation is greater control of the well. If milling or reaming is performed the score is 1, if the well is perforated, it scores with 0.5, if not milled, reamed or perforated the score is 0.

### 1.12 Secondary indication of well leakage (*sc_ind_lk*)

The possibility of leakage through a well or along a well path can be indicated by secondary measurements such as gas leakage to seafloor [2,3] or detailed studies of pockmark development associated with shallow gas leakage [11]. Up to now, such measurements are rarely available, but we expect a rapid increase if monitoring of P&A'ed wells will be required or abandoned wells will be re-visited and re-evaluated. If leakage is indicated the well gets a score of 0. More than 7000 pockmarks are mapped in the Troll East area and geochemical and petrographic results indicate that these pockmarks formed as a consequence of rapid climatic changes following the Younger Dryas and are not active at present [11]. Thus, all wells from Troll East score with 2. Wells from Troll West score with 1 (no secondary tests performed).

### 1.13 Total P&A score (*sc_total*)

**Fig. 2:**
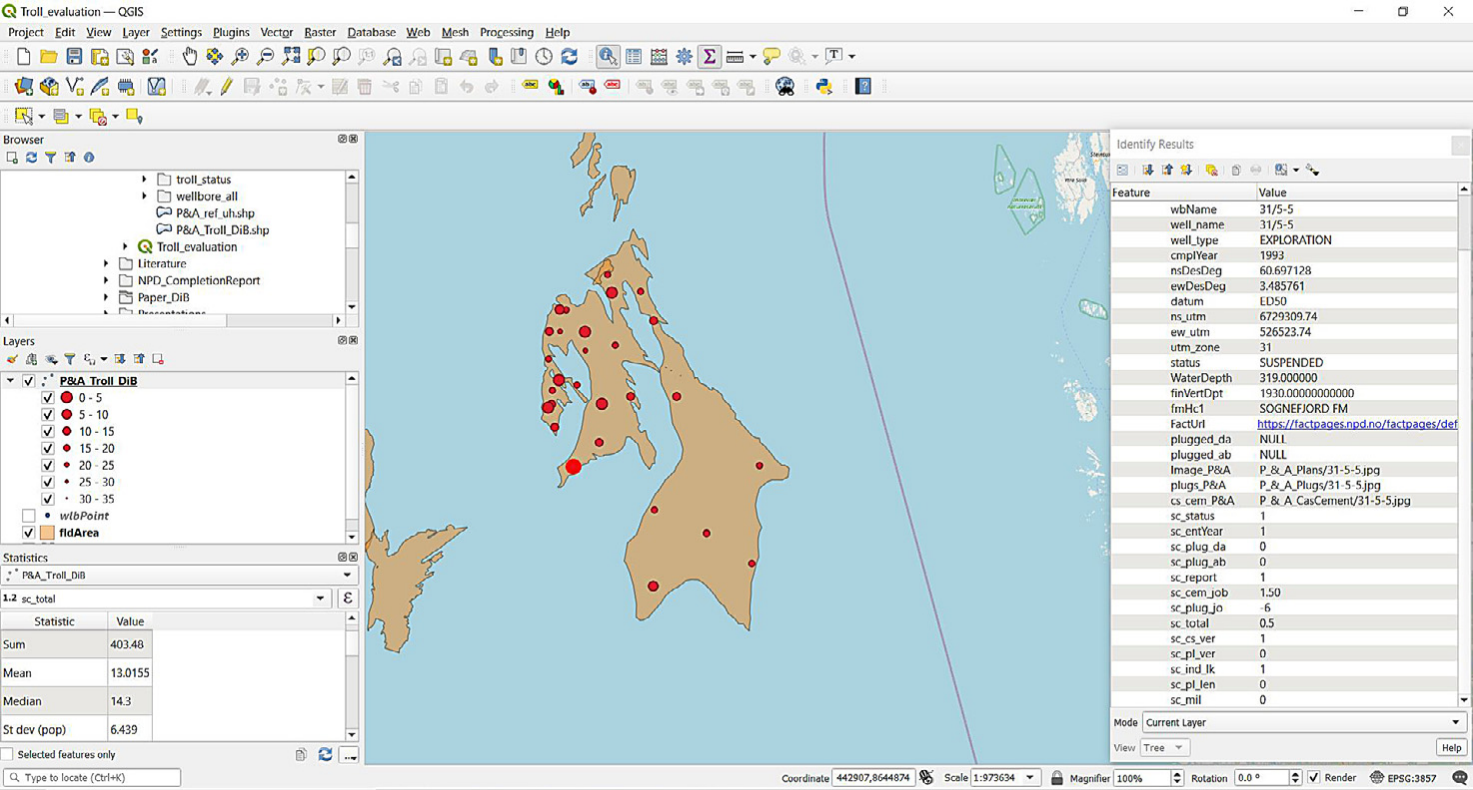
Screenshot of the QGIS interface [6] showing the locations of the evaluated well bores (red dots) within the Troll gas and oil field (brownish background) offshore SW Norway. The dot size is related to the total score numbers (shown in the *Layers* panel). The location of well 31/5–5 is highlighted, and the individual field values can be analysed in the *Identify Results* panel. The QGIS project with associated data is provided in the supplementary data (*Troll_P&A*).

The sum of all scores and can vary from −6 to +30. A summary of all scored wells in the Troll area is displayed in the QGIS software screenshot of Fig. 2.

## 2 Experimental Design, Materials and Methods

We developed a scoring system to evaluate the P&A quality of exploration wells and applied this method to wells from the Troll gas and oil field in the Norwegian Sea. The analysis is based on primary data extracted from the website of the NPD and mined from well completion reports (https://factpages.npd.no/en/wellbore). The raw data contains e.g., date of drilling, date of reporting towards the authorities, status, hole sizes, casing sizes, vertical depth, plug sizes, plug vertical depth, amount of used cement, applied verification tests. Usually, all information is given in the drilling or operation sections of the well completion reports, reported in the summary for the different hole/casing intervals, the description of the daily drilling/operation, given in tables and/or shown in schematic drawings.

The extracted primary data is then evaluated by different criteria described in the data description section. The evaluation results are implemented into an QGIS environment [6] for fast access and assessment (provided in the supplementary data as a shape file). The final total score gives a status of the reporting scheme, and an individual assessment of the P&A quality but it does not provide e.g., a leaking risk probability for the analysed wells. Table 1

## Ethics Statement

 

## CRediT Author Statement

**Benjamin Emmel:** Conceptualization, Methodology, Software, Data curation, Writing, Visualization. **Bastien Dupuy:** Writing – Reviewing and Editing

## Declaration of competing interest

The authors declare that they have no known competing financial interests or personal relationships which have or could be perceived to have influenced the work reported in this article.

## Appendix Supplementary materials

Image, application 1
