# Supplementary figures and images for: Dataset of plugging and abandonment status from exploration wells drilled within the Troll gas and oil field in the Norwegian North Sea
Source: Data Brief. 2021 May 25;37:107165. doi: 10.1016/j.dib.2021.107165 (PMC8181855; doi:10.1016/j.dib.2021.107165)

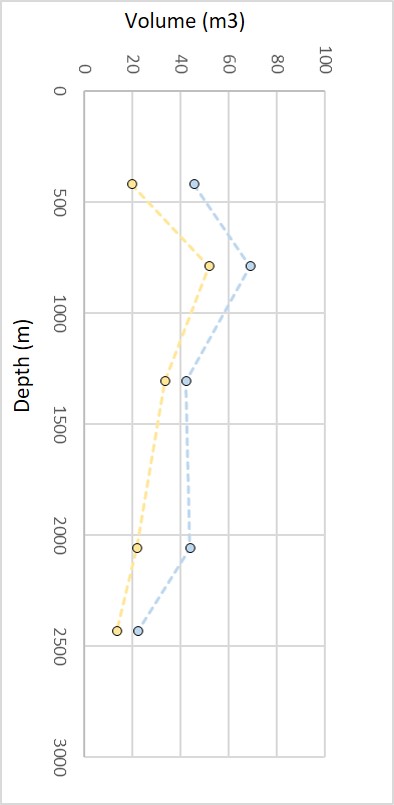

Supplement: Supplementary file 1 [file mmc1.zip › Data_P&A/P_&_A_CasCement/31-2-1.jpg]

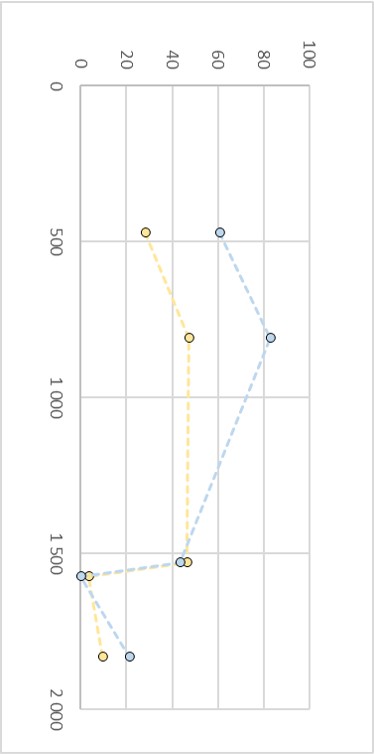

Supplement: Supplementary file 1 [file mmc1.zip › Data_P&A/P_&_A_CasCement/31-2-10.jpg]

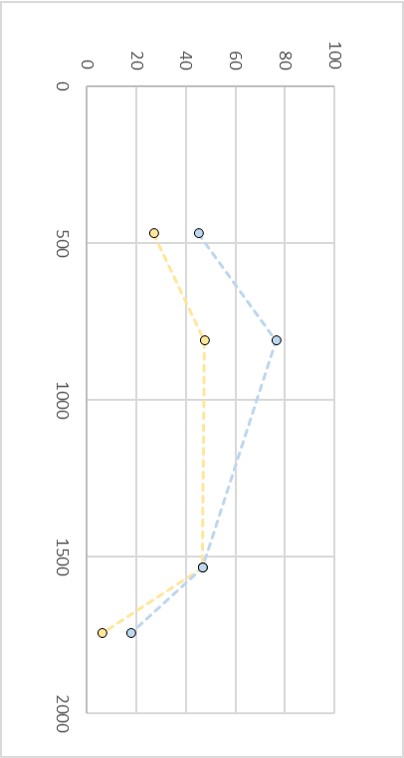

Supplement: Supplementary file 1 [file mmc1.zip › Data_P&A/P_&_A_CasCement/31-2-11.jpg]

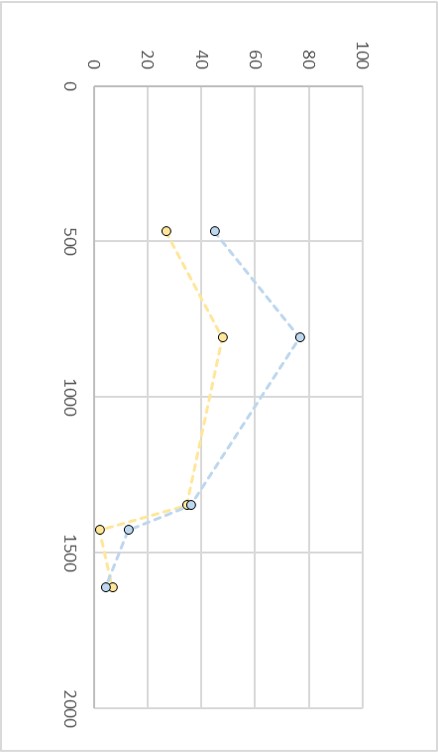

Supplement: Supplementary file 1 [file mmc1.zip › Data_P&A/P_&_A_CasCement/31-2-12.jpg]

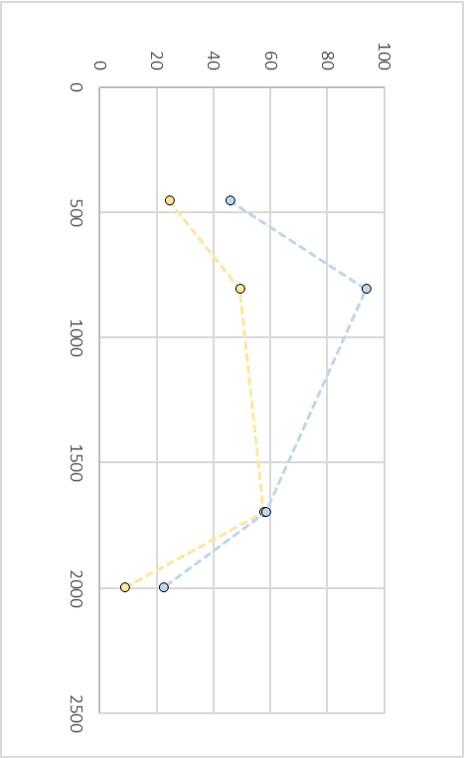

Supplement: Supplementary file 1 [file mmc1.zip › Data_P&A/P_&_A_CasCement/31-2-13S.jpg]

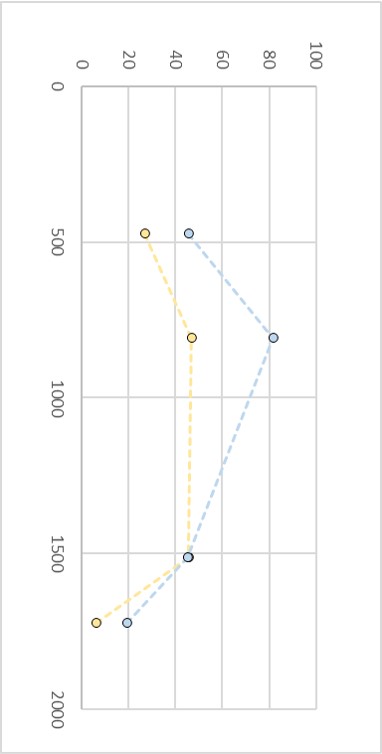

Supplement: Supplementary file 1 [file mmc1.zip › Data_P&A/P_&_A_CasCement/31-2-14.jpg]

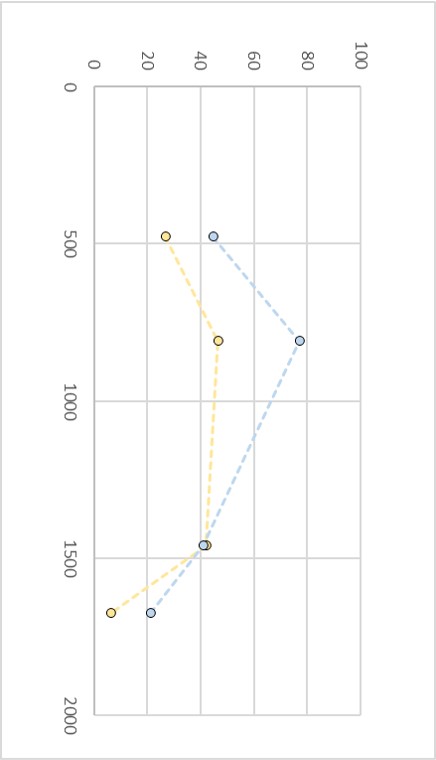

Supplement: Supplementary file 1 [file mmc1.zip › Data_P&A/P_&_A_CasCement/31-2-15.jpg]

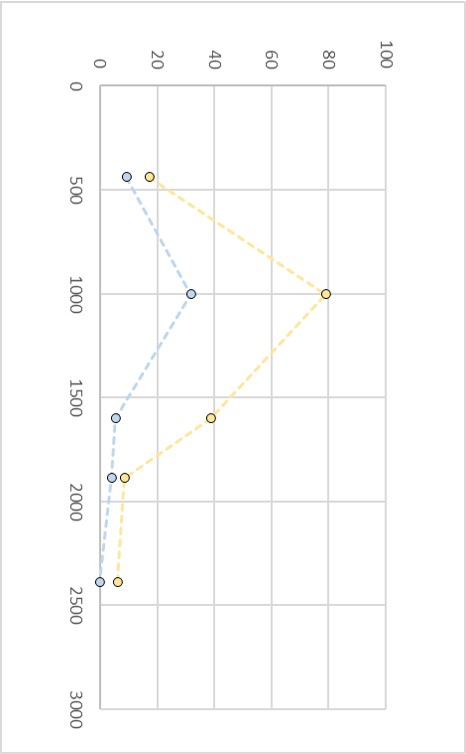

Supplement: Supplementary file 1 [file mmc1.zip › Data_P&A/P_&_A_CasCement/31-2-16S.jpg]

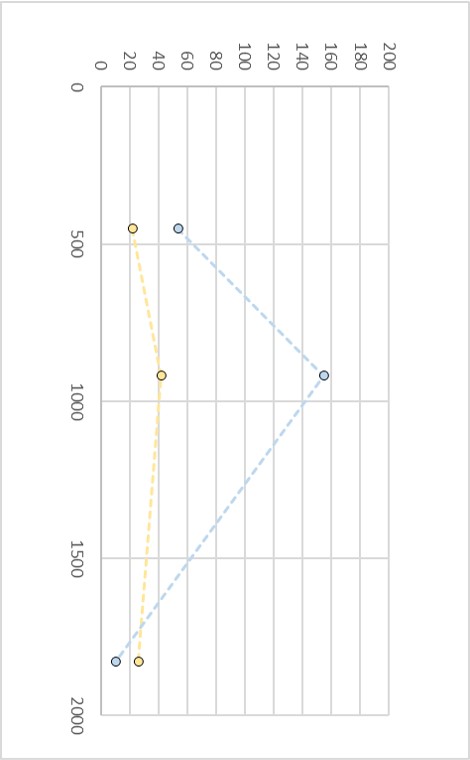

Supplement: Supplementary file 1 [file mmc1.zip › Data_P&A/P_&_A_CasCement/31-2-17S.jpg]

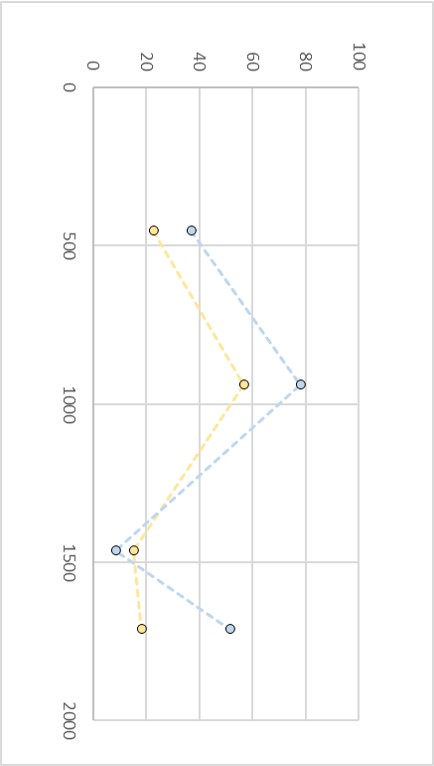

Supplement: Supplementary file 1 [file mmc1.zip › Data_P&A/P_&_A_CasCement/31-2-18.jpg]

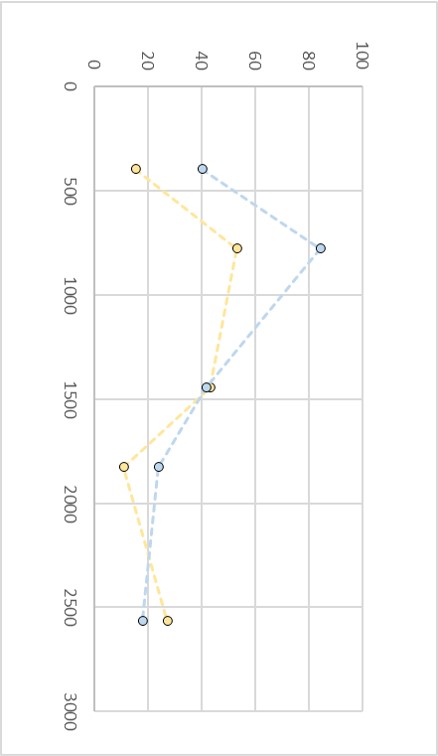

Supplement: Supplementary file 1 [file mmc1.zip › Data_P&A/P_&_A_CasCement/31-2-2.jpg]

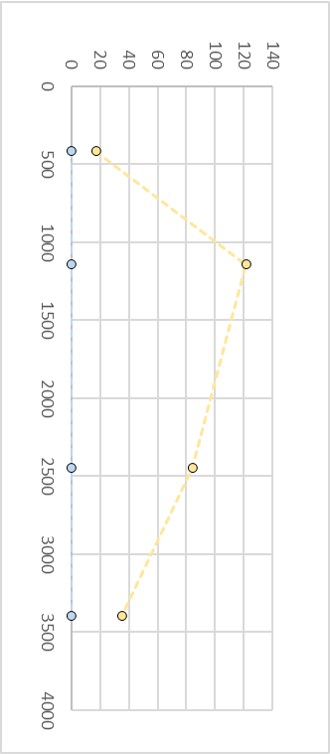

Supplement: Supplementary file 1 [file mmc1.zip › Data_P&A/P_&_A_CasCement/31-2-20S.jpg]

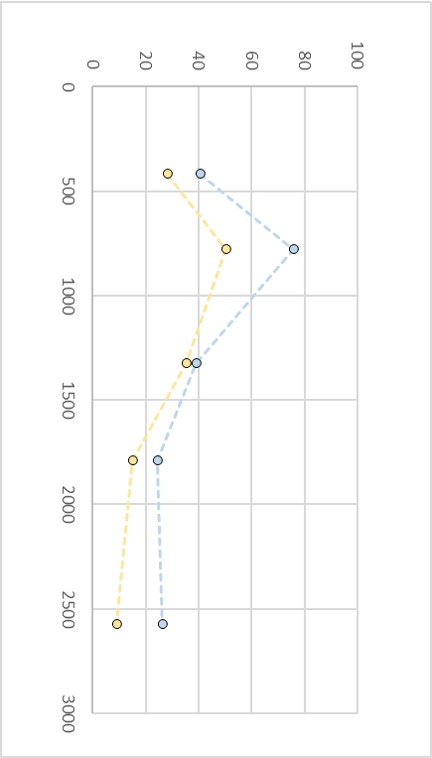

Supplement: Supplementary file 1 [file mmc1.zip › Data_P&A/P_&_A_CasCement/31-2-3.jpg]

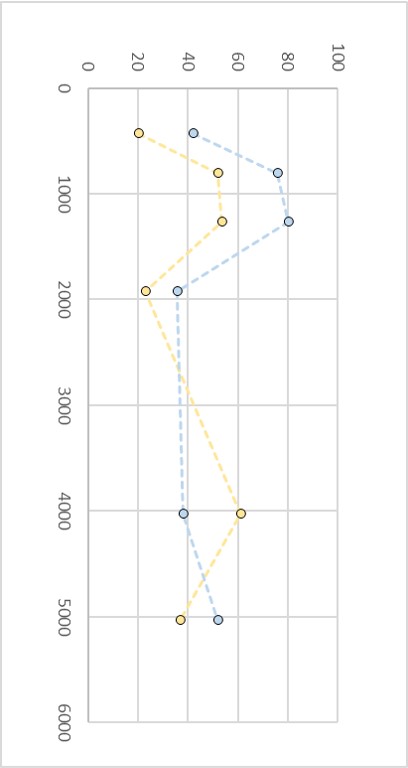

Supplement: Supplementary file 1 [file mmc1.zip › Data_P&A/P_&_A_CasCement/31-2-4.jpg]

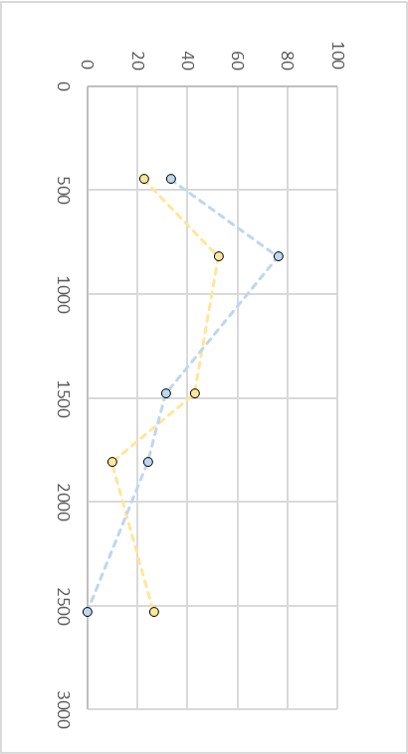

Supplement: Supplementary file 1 [file mmc1.zip › Data_P&A/P_&_A_CasCement/31-2-5.jpg]

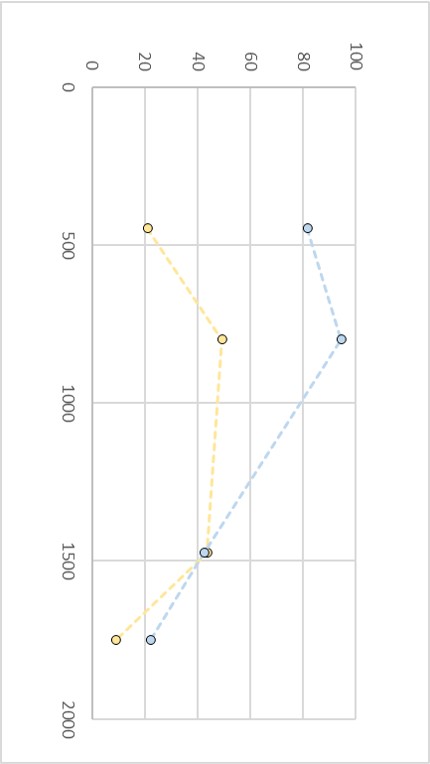

Supplement: Supplementary file 1 [file mmc1.zip › Data_P&A/P_&_A_CasCement/31-2-6.jpg]

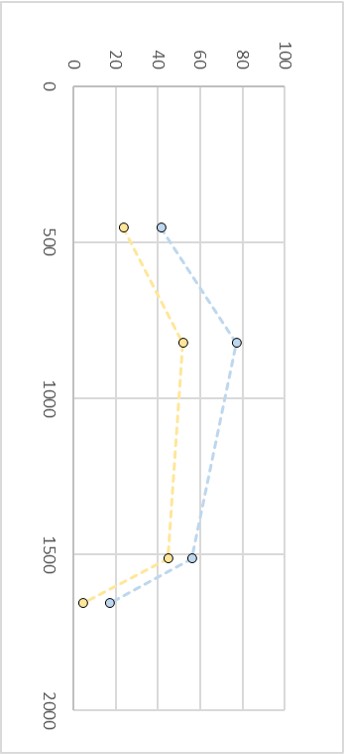

Supplement: Supplementary file 1 [file mmc1.zip › Data_P&A/P_&_A_CasCement/31-2-7.jpg]

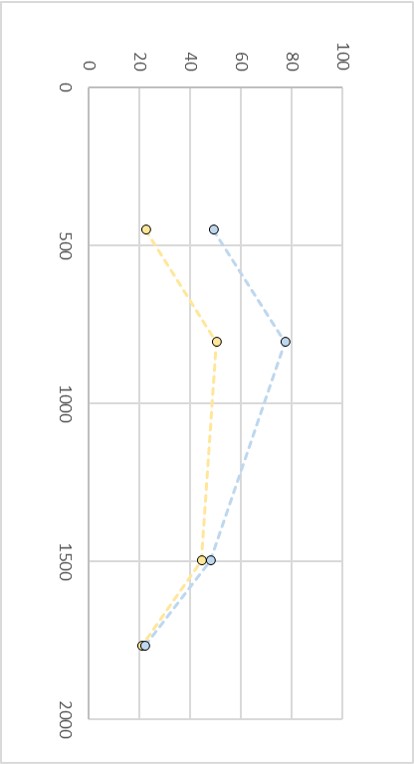

Supplement: Supplementary file 1 [file mmc1.zip › Data_P&A/P_&_A_CasCement/31-2-9.jpg]

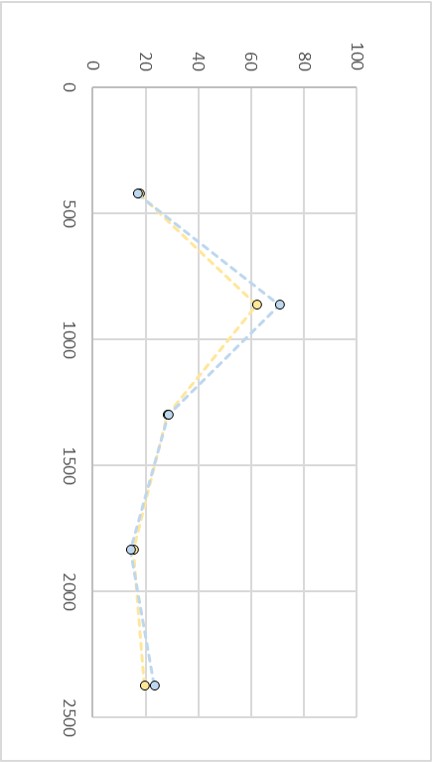

Supplement: Supplementary file 1 [file mmc1.zip › Data_P&A/P_&_A_CasCement/31-3-1.jpg]

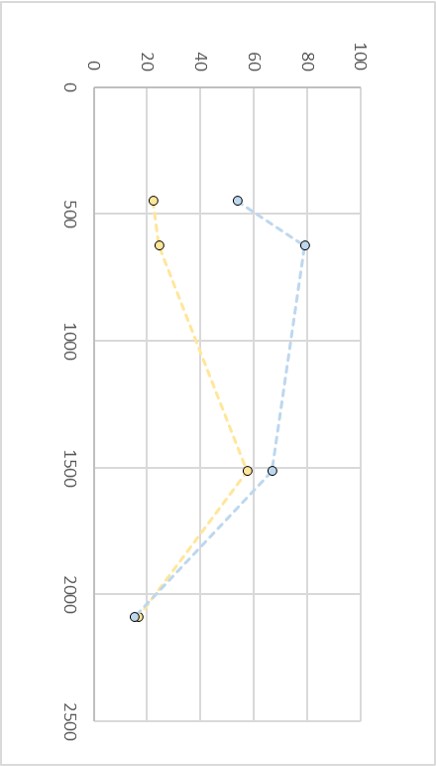

Supplement: Supplementary file 1 [file mmc1.zip › Data_P&A/P_&_A_CasCement/31-3-2.jpg]

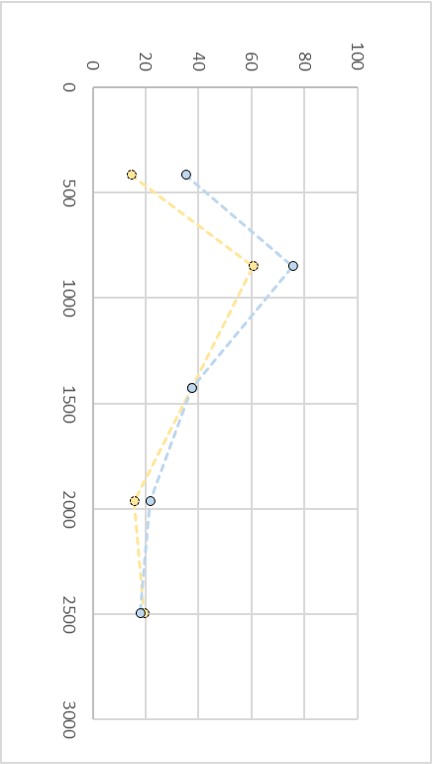

Supplement: Supplementary file 1 [file mmc1.zip › Data_P&A/P_&_A_CasCement/31-5-2.jpg]

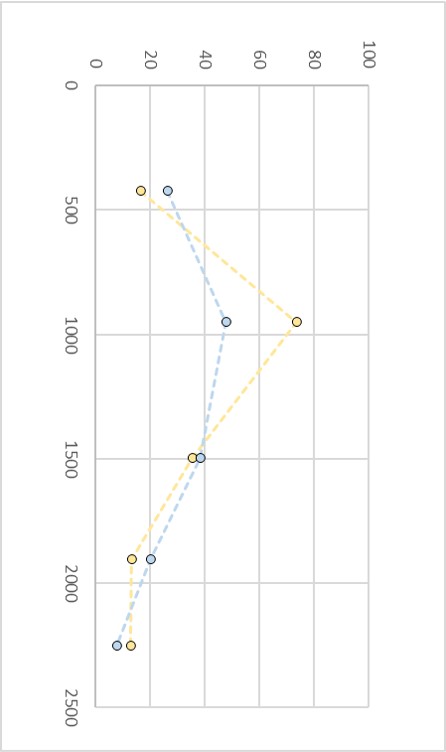

Supplement: Supplementary file 1 [file mmc1.zip › Data_P&A/P_&_A_CasCement/31-5-3.jpg]

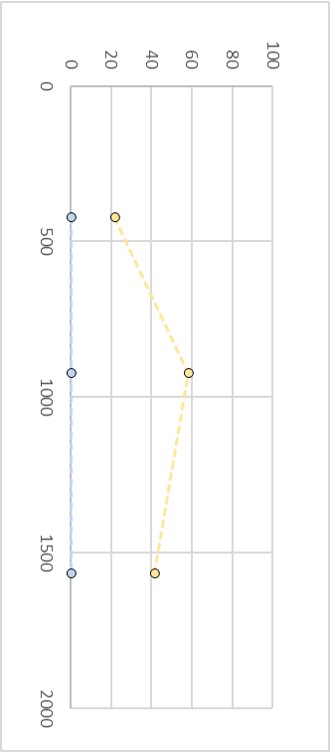

Supplement: Supplementary file 1 [file mmc1.zip › Data_P&A/P_&_A_CasCement/31-5-4.jpg]

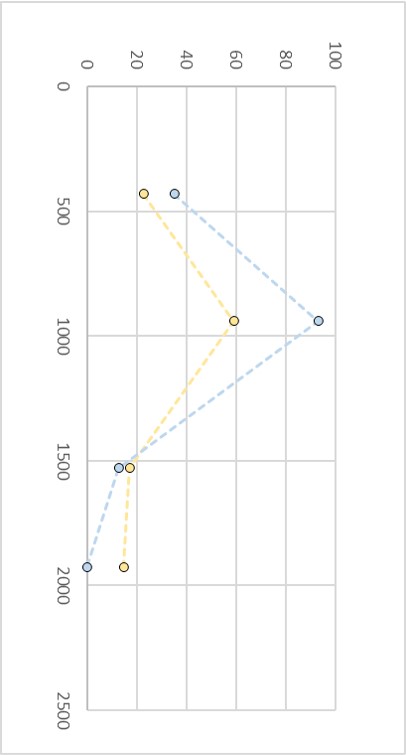

Supplement: Supplementary file 1 [file mmc1.zip › Data_P&A/P_&_A_CasCement/31-5-5.jpg]

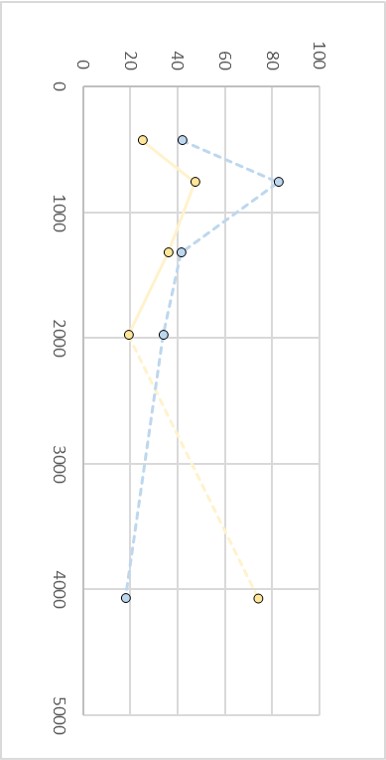

Supplement: Supplementary file 1 [file mmc1.zip › Data_P&A/P_&_A_CasCement/31-6-1.jpg]

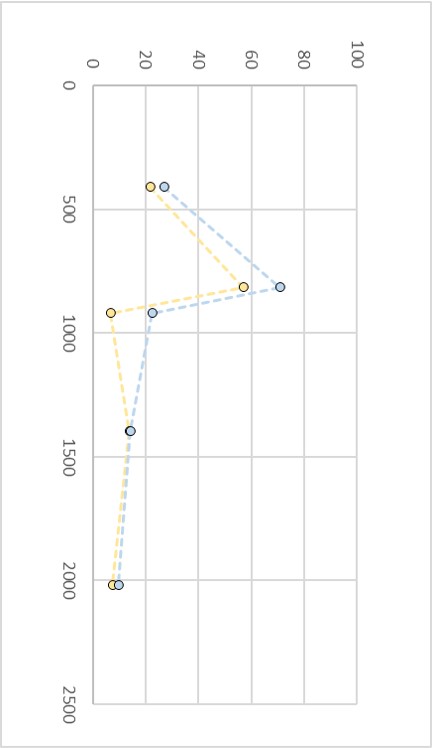

Supplement: Supplementary file 1 [file mmc1.zip › Data_P&A/P_&_A_CasCement/31-6-2.jpg]

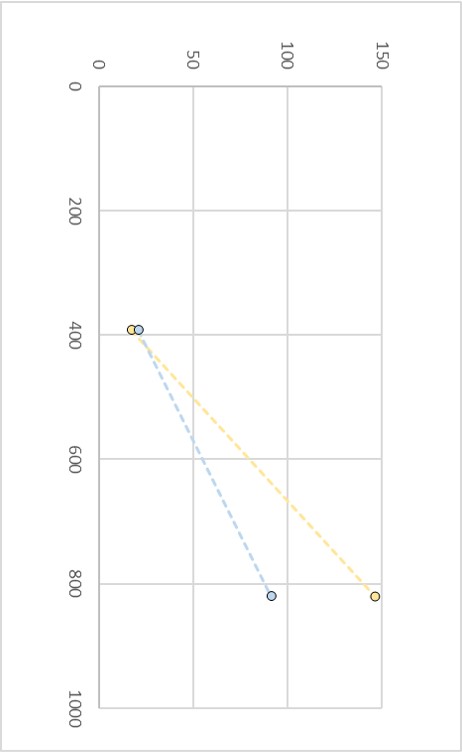

Supplement: Supplementary file 1 [file mmc1.zip › Data_P&A/P_&_A_CasCement/31-6-4.jpg]

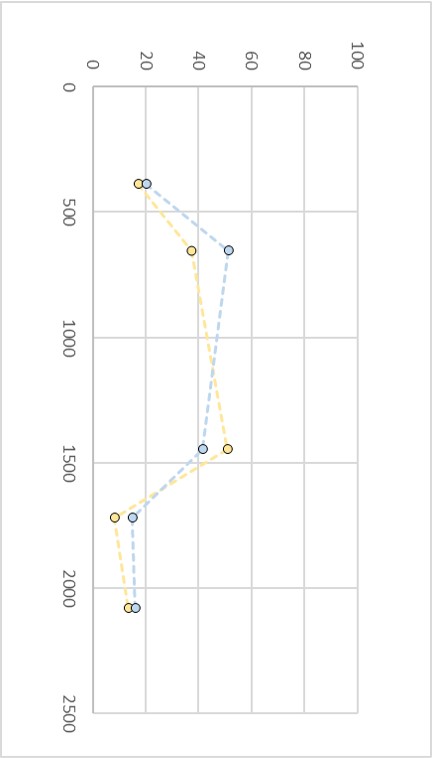

Supplement: Supplementary file 1 [file mmc1.zip › Data_P&A/P_&_A_CasCement/31-6-5.jpg]

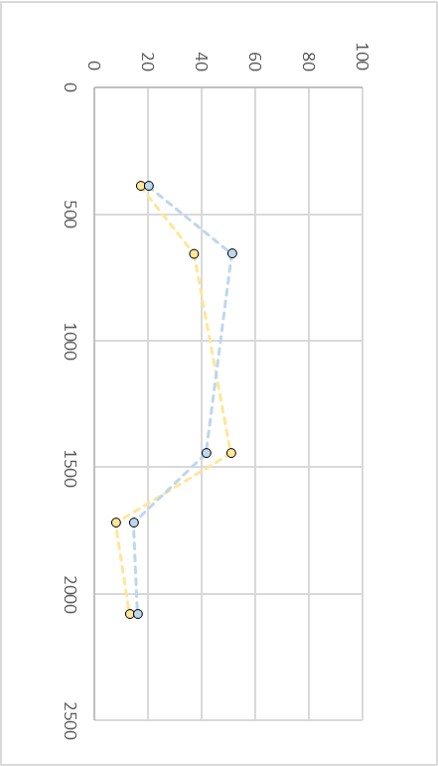

Supplement: Supplementary file 1 [file mmc1.zip › Data_P&A/P_&_A_CasCement/31-6-6.jpg]

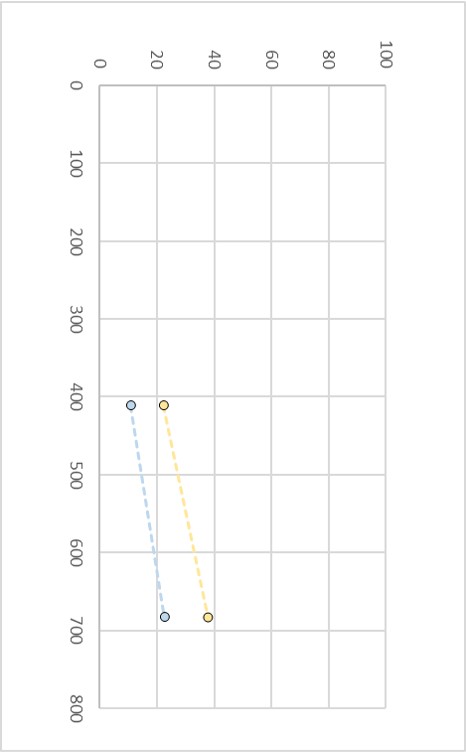

Supplement: Supplementary file 1 [file mmc1.zip › Data_P&A/P_&_A_CasCement/31-6-7.jpg]

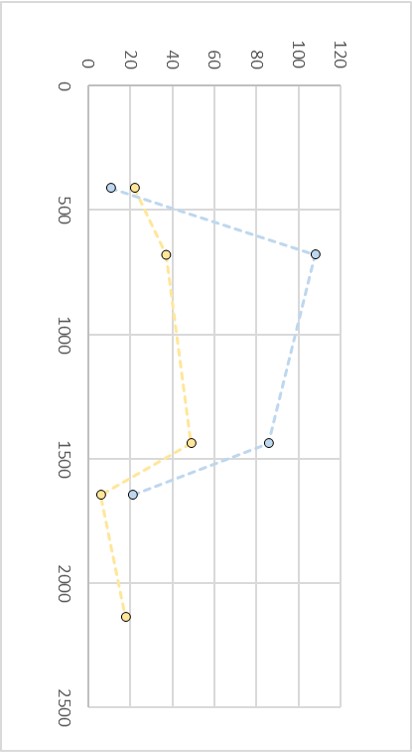

Supplement: Supplementary file 1 [file mmc1.zip › Data_P&A/P_&_A_CasCement/31-6-8.jpg]

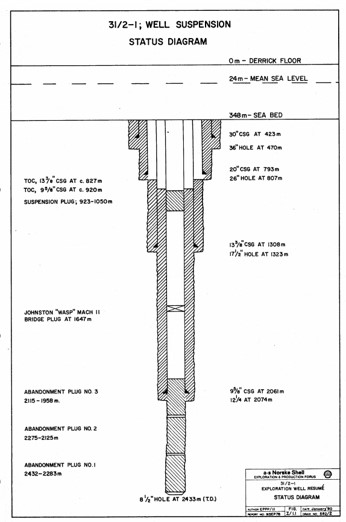

Supplement: Supplementary file 1 [file mmc1.zip › Data_P&A/P_&_A_Plans/31-2-1.jpg]

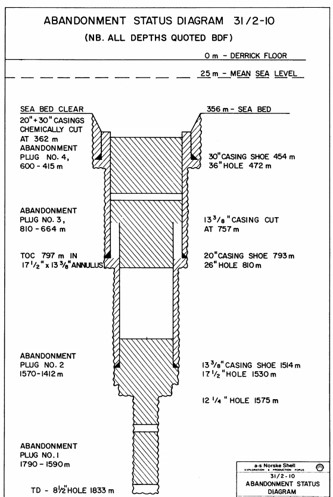

Supplement: Supplementary file 1 [file mmc1.zip › Data_P&A/P_&_A_Plans/31-2-10.jpg]

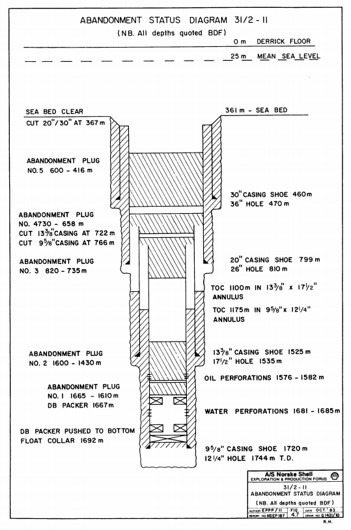

Supplement: Supplementary file 1 [file mmc1.zip › Data_P&A/P_&_A_Plans/31-2-11.jpg]

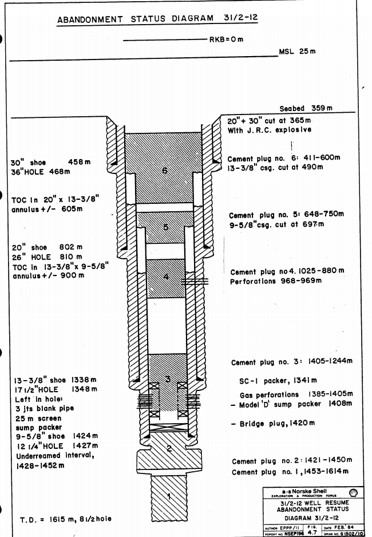

Supplement: Supplementary file 1 [file mmc1.zip › Data_P&A/P_&_A_Plans/31-2-12.jpg]

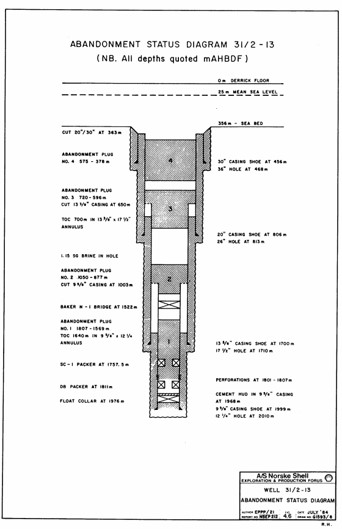

Supplement: Supplementary file 1 [file mmc1.zip › Data_P&A/P_&_A_Plans/31-2-13S.jpg]

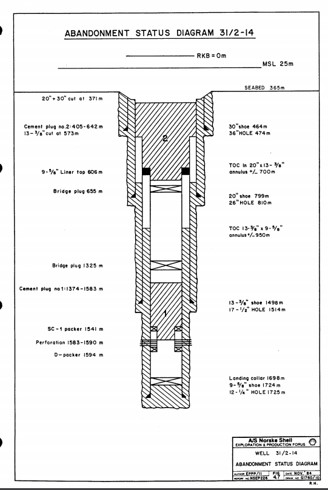

Supplement: Supplementary file 1 [file mmc1.zip › Data_P&A/P_&_A_Plans/31-2-14.jpg]

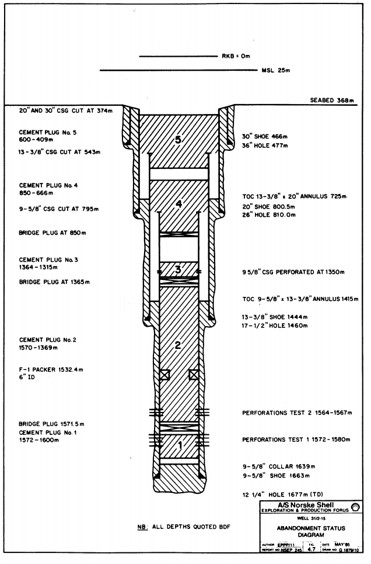

Supplement: Supplementary file 1 [file mmc1.zip › Data_P&A/P_&_A_Plans/31-2-15.jpg]

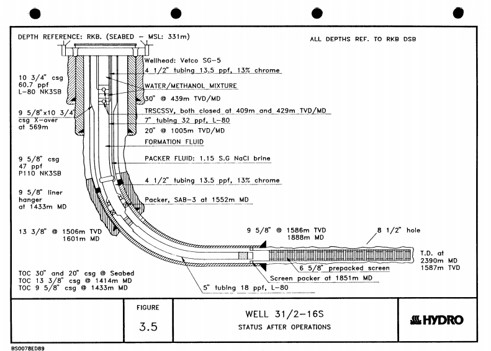

Supplement: Supplementary file 1 [file mmc1.zip › Data_P&A/P_&_A_Plans/31-2-16S.jpg]

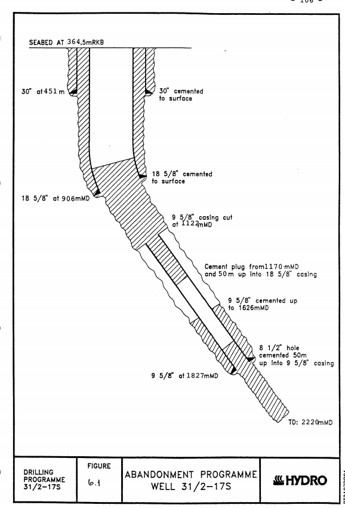

Supplement: Supplementary file 1 [file mmc1.zip › Data_P&A/P_&_A_Plans/31-2-17S.jpg]

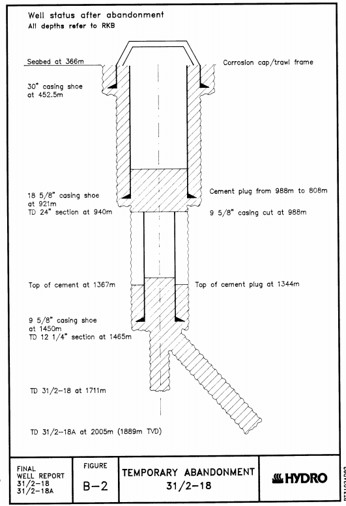

Supplement: Supplementary file 1 [file mmc1.zip › Data_P&A/P_&_A_Plans/31-2-18.jpg]

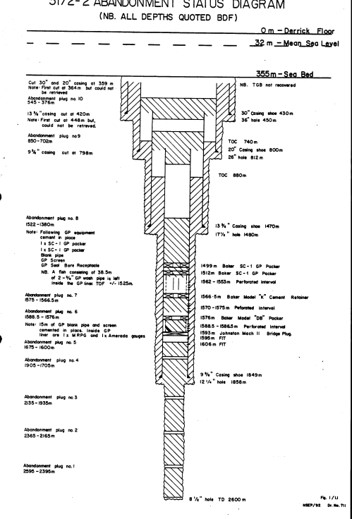

Supplement: Supplementary file 1 [file mmc1.zip › Data_P&A/P_&_A_Plans/31-2-2.jpg]

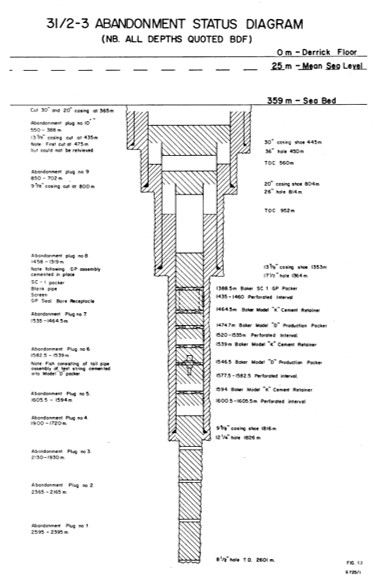

Supplement: Supplementary file 1 [file mmc1.zip › Data_P&A/P_&_A_Plans/31-2-3.jpg]

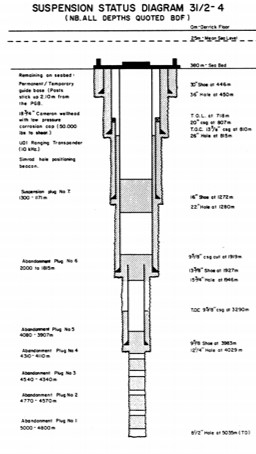

Supplement: Supplementary file 1 [file mmc1.zip › Data_P&A/P_&_A_Plans/31-2-4.jpg]

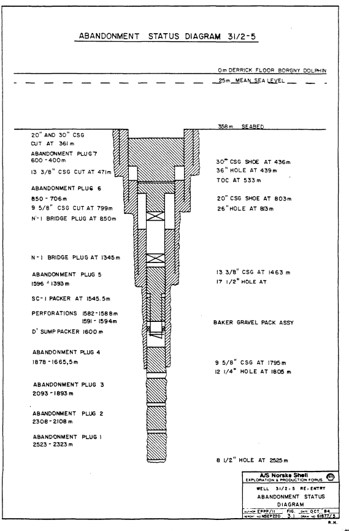

Supplement: Supplementary file 1 [file mmc1.zip › Data_P&A/P_&_A_Plans/31-2-5.jpg]

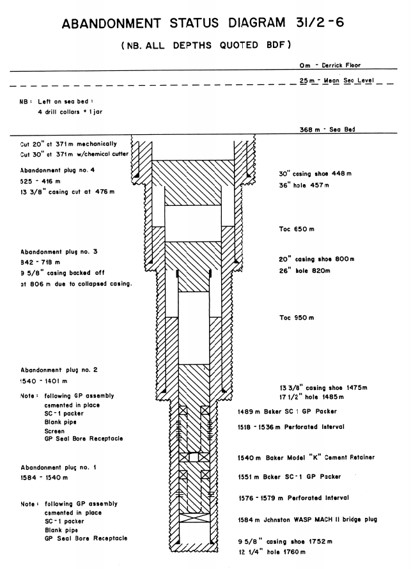

Supplement: Supplementary file 1 [file mmc1.zip › Data_P&A/P_&_A_Plans/31-2-6.jpg]

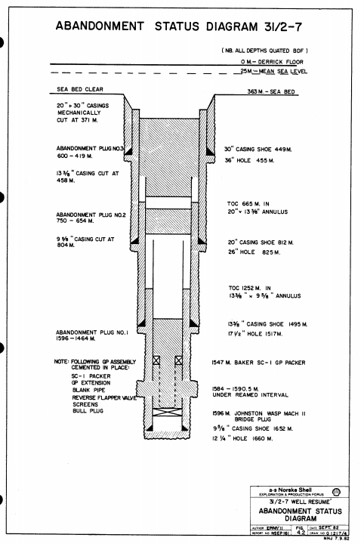

Supplement: Supplementary file 1 [file mmc1.zip › Data_P&A/P_&_A_Plans/31-2-7.jpg]

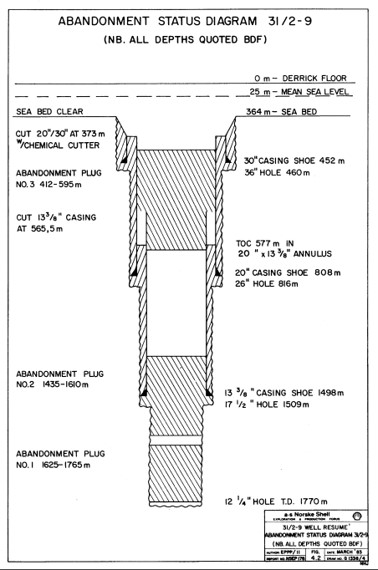

Supplement: Supplementary file 1 [file mmc1.zip › Data_P&A/P_&_A_Plans/31-2-9.jpg]

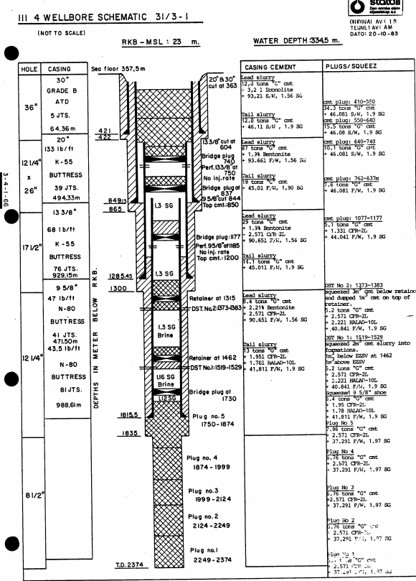

Supplement: Supplementary file 1 [file mmc1.zip › Data_P&A/P_&_A_Plans/31-3-1.jpg]

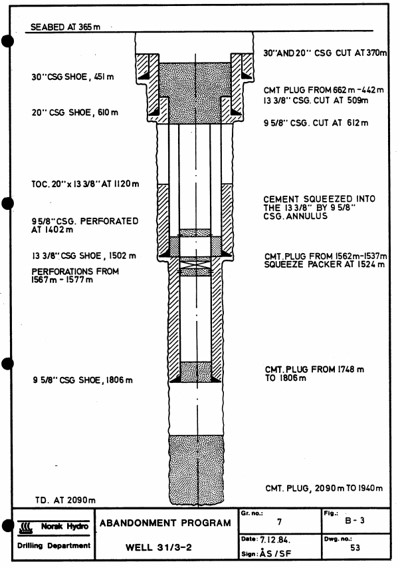

Supplement: Supplementary file 1 [file mmc1.zip › Data_P&A/P_&_A_Plans/31-3-2.jpg]

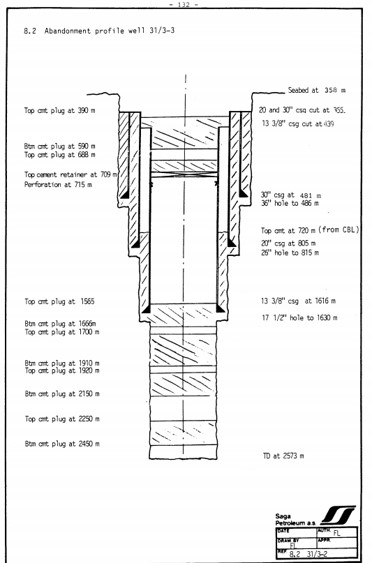

Supplement: Supplementary file 1 [file mmc1.zip › Data_P&A/P_&_A_Plans/31-3-3.jpg]

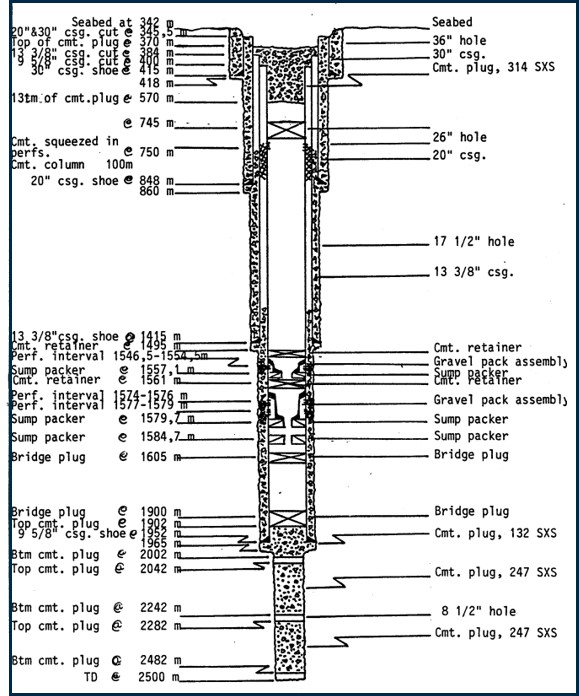

Supplement: Supplementary file 1 [file mmc1.zip › Data_P&A/P_&_A_Plans/31-5-2.jpg]

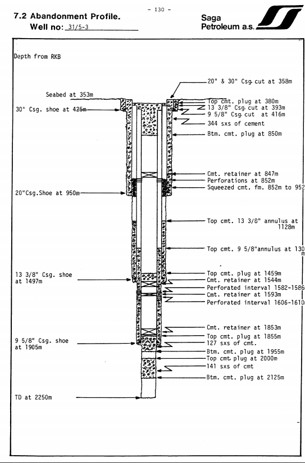

Supplement: Supplementary file 1 [file mmc1.zip › Data_P&A/P_&_A_Plans/31-5-3.jpg]

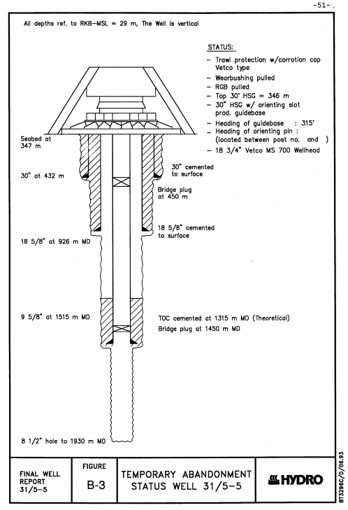

Supplement: Supplementary file 1 [file mmc1.zip › Data_P&A/P_&_A_Plans/31-5-5.jpg]

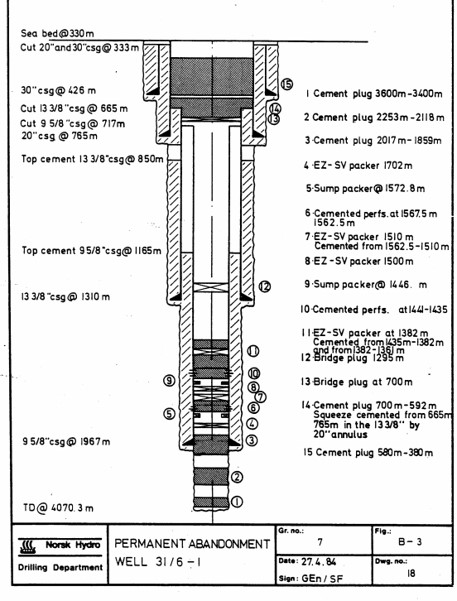

Supplement: Supplementary file 1 [file mmc1.zip › Data_P&A/P_&_A_Plans/31-6-1.jpg]

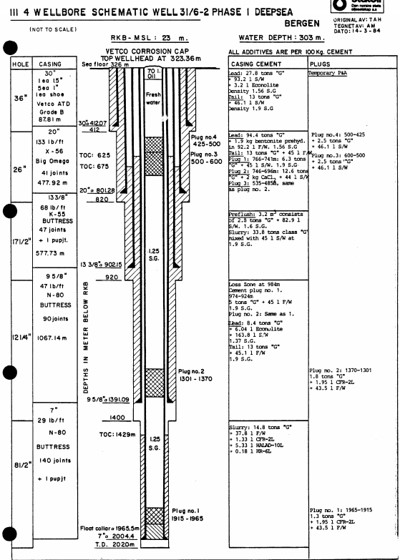

Supplement: Supplementary file 1 [file mmc1.zip › Data_P&A/P_&_A_Plans/31-6-2.jpg]

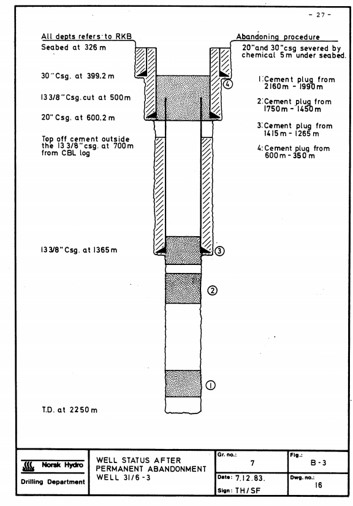

Supplement: Supplementary file 1 [file mmc1.zip › Data_P&A/P_&_A_Plans/31-6-3.jpg]

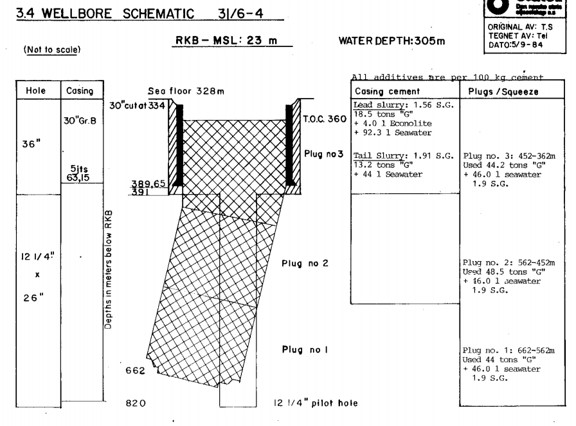

Supplement: Supplementary file 1 [file mmc1.zip › Data_P&A/P_&_A_Plans/31-6-4.jpg]

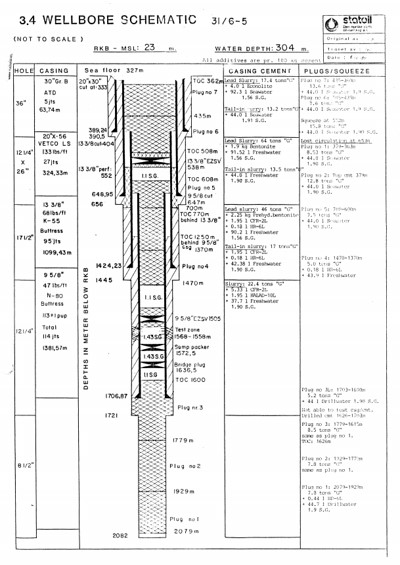

Supplement: Supplementary file 1 [file mmc1.zip › Data_P&A/P_&_A_Plans/31-6-5.jpg]

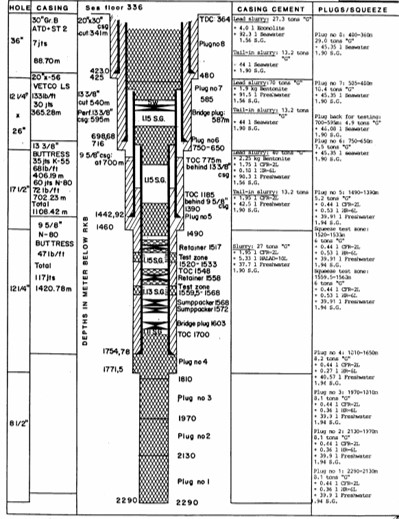

Supplement: Supplementary file 1 [file mmc1.zip › Data_P&A/P_&_A_Plans/31-6-6.jpg]

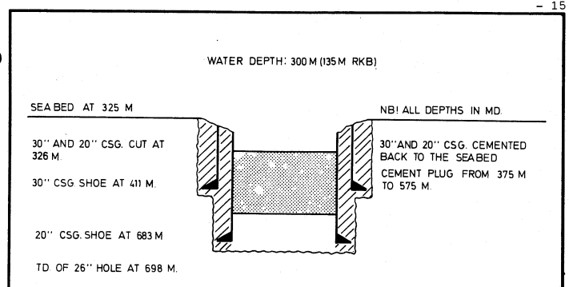

Supplement: Supplementary file 1 [file mmc1.zip › Data_P&A/P_&_A_Plans/31-6-7.jpg]

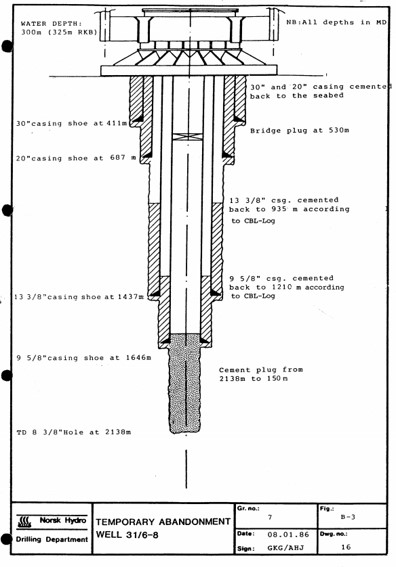

Supplement: Supplementary file 1 [file mmc1.zip › Data_P&A/P_&_A_Plans/31-6-8.jpg]

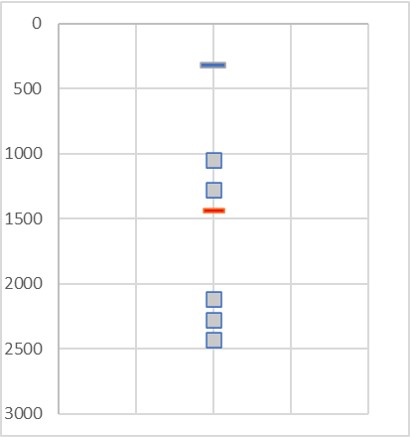

Supplement: Supplementary file 1 [file mmc1.zip › Data_P&A/P_&_A_Plugs/31-2-1.jpg]

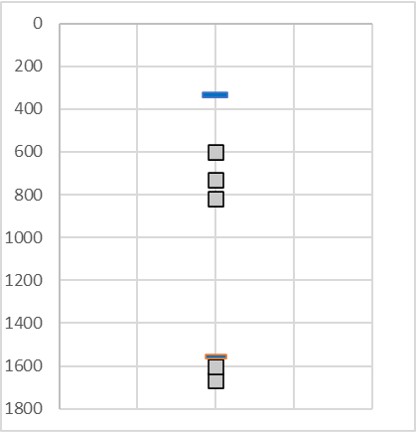

Supplement: Supplementary file 1 [file mmc1.zip › Data_P&A/P_&_A_Plugs/31-2-11.jpg]

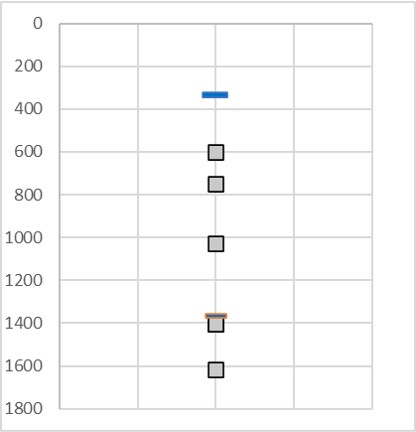

Supplement: Supplementary file 1 [file mmc1.zip › Data_P&A/P_&_A_Plugs/31-2-12.jpg]

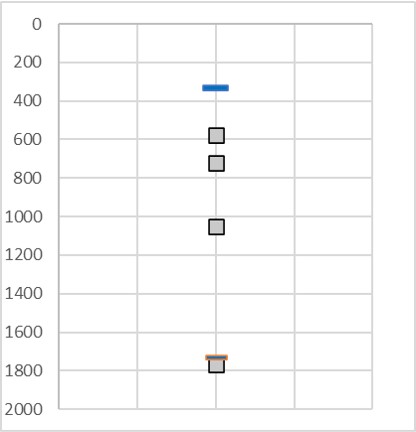

Supplement: Supplementary file 1 [file mmc1.zip › Data_P&A/P_&_A_Plugs/31-2-13S.jpg]

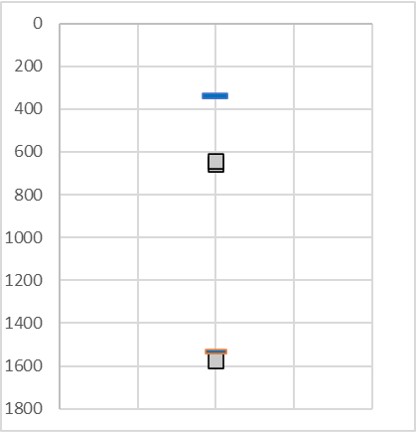

Supplement: Supplementary file 1 [file mmc1.zip › Data_P&A/P_&_A_Plugs/31-2-14.jpg]

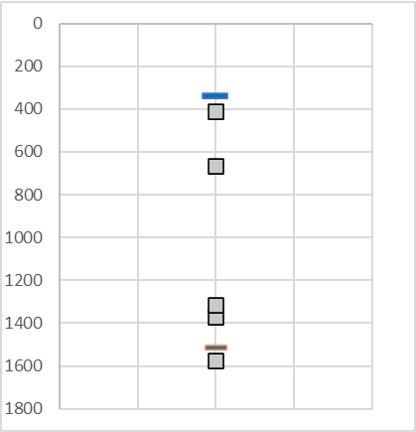

Supplement: Supplementary file 1 [file mmc1.zip › Data_P&A/P_&_A_Plugs/31-2-15.jpg]

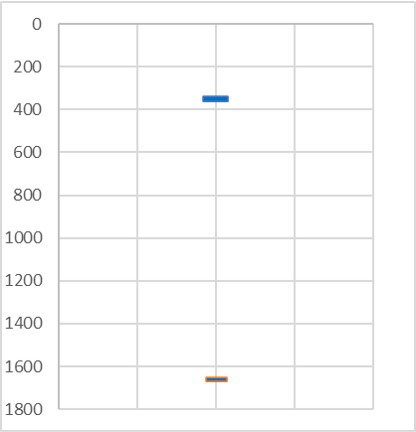

Supplement: Supplementary file 1 [file mmc1.zip › Data_P&A/P_&_A_Plugs/31-2-16S.jpg]

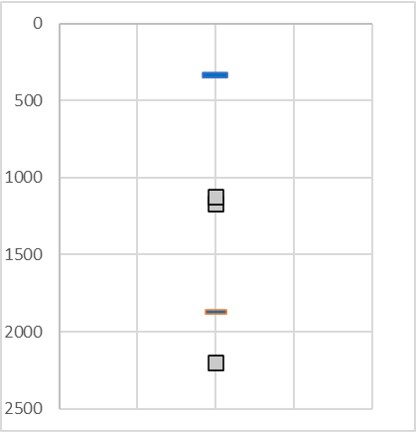

Supplement: Supplementary file 1 [file mmc1.zip › Data_P&A/P_&_A_Plugs/31-2-17S.jpg]

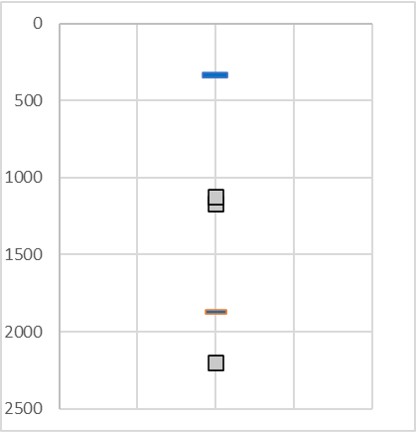

Supplement: Supplementary file 1 [file mmc1.zip › Data_P&A/P_&_A_Plugs/31-2-18.jpg]

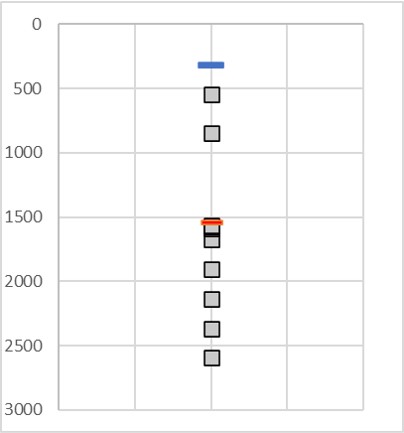

Supplement: Supplementary file 1 [file mmc1.zip › Data_P&A/P_&_A_Plugs/31-2-2.jpg]

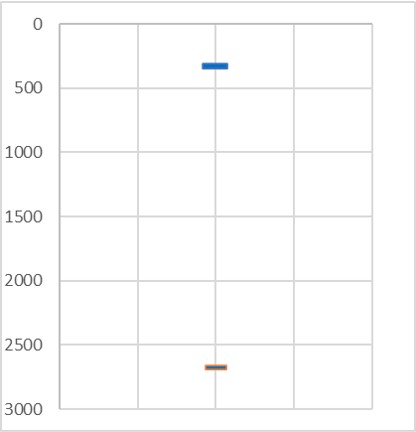

Supplement: Supplementary file 1 [file mmc1.zip › Data_P&A/P_&_A_Plugs/31-2-20S.jpg]

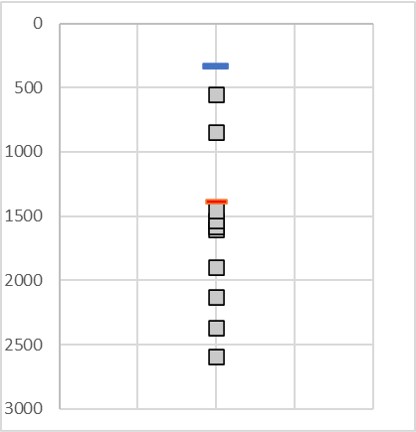

Supplement: Supplementary file 1 [file mmc1.zip › Data_P&A/P_&_A_Plugs/31-2-3.jpg]

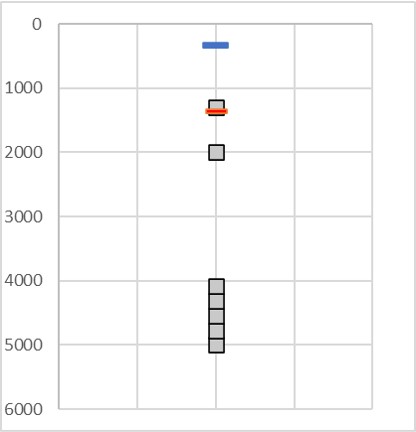

Supplement: Supplementary file 1 [file mmc1.zip › Data_P&A/P_&_A_Plugs/31-2-4.jpg]

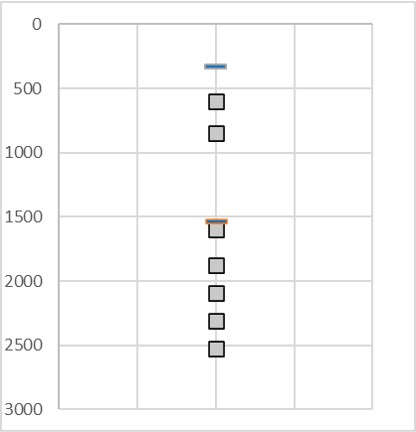

Supplement: Supplementary file 1 [file mmc1.zip › Data_P&A/P_&_A_Plugs/31-2-5.jpg]

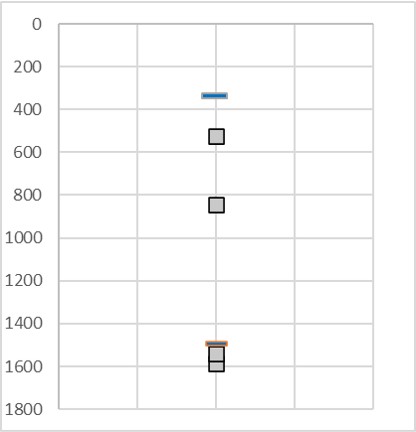

Supplement: Supplementary file 1 [file mmc1.zip › Data_P&A/P_&_A_Plugs/31-2-6.jpg]

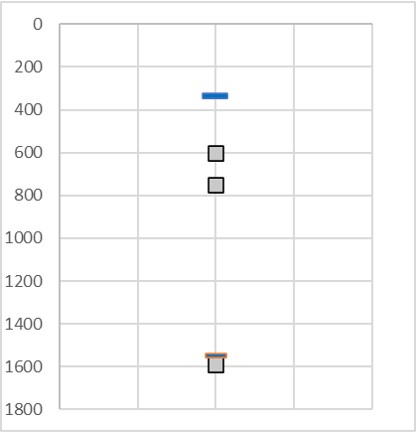

Supplement: Supplementary file 1 [file mmc1.zip › Data_P&A/P_&_A_Plugs/31-2-7.jpg]

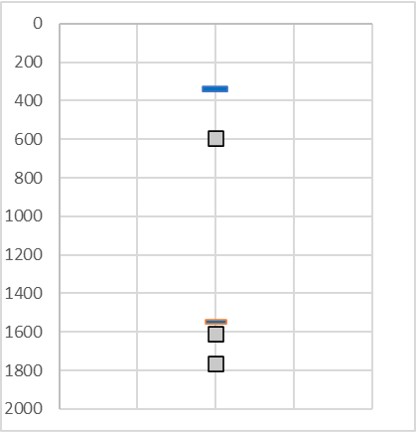

Supplement: Supplementary file 1 [file mmc1.zip › Data_P&A/P_&_A_Plugs/31-2-9.jpg]

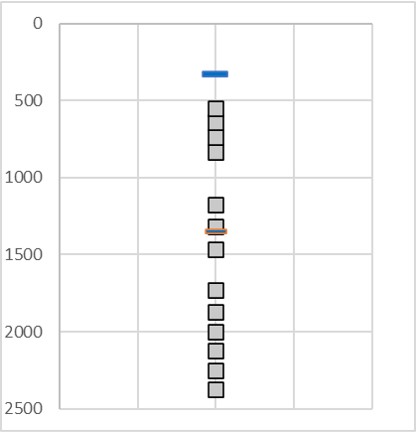

Supplement: Supplementary file 1 [file mmc1.zip › Data_P&A/P_&_A_Plugs/31-3-1.jpg]

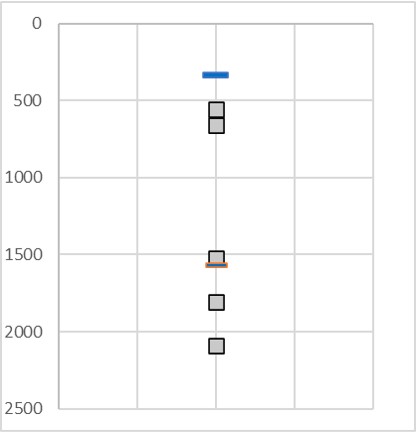

Supplement: Supplementary file 1 [file mmc1.zip › Data_P&A/P_&_A_Plugs/31-3-2.jpg]

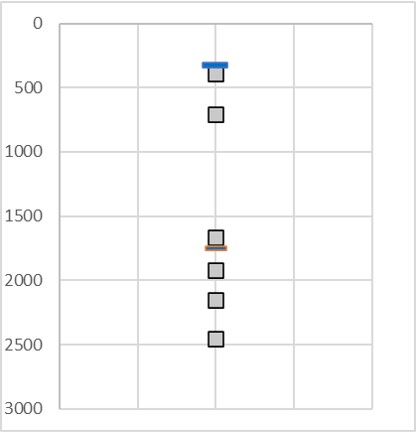

Supplement: Supplementary file 1 [file mmc1.zip › Data_P&A/P_&_A_Plugs/31-3-3.jpg]

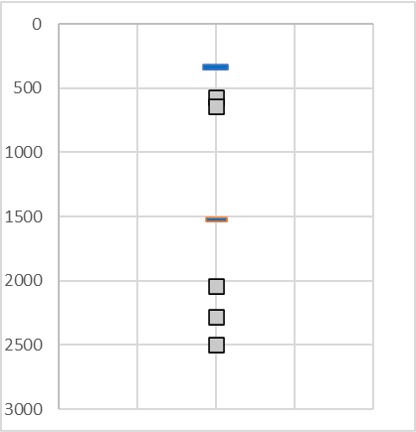

Supplement: Supplementary file 1 [file mmc1.zip › Data_P&A/P_&_A_Plugs/31-5-2.jpg]

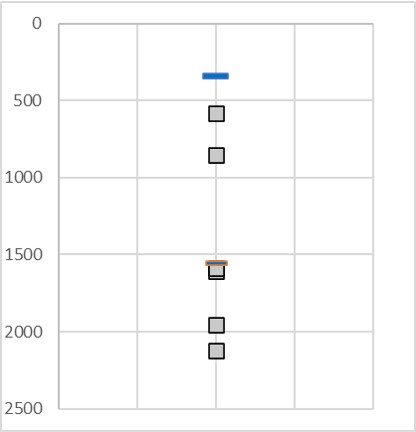

Supplement: Supplementary file 1 [file mmc1.zip › Data_P&A/P_&_A_Plugs/31-5-3.jpg]

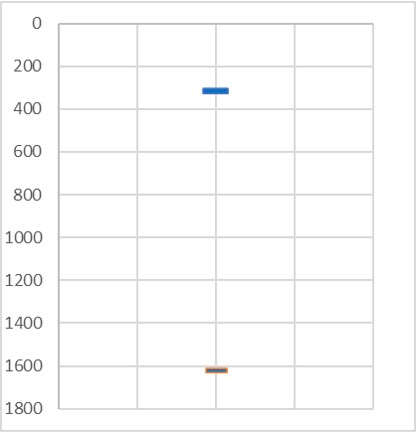

Supplement: Supplementary file 1 [file mmc1.zip › Data_P&A/P_&_A_Plugs/31-5-4.jpg]

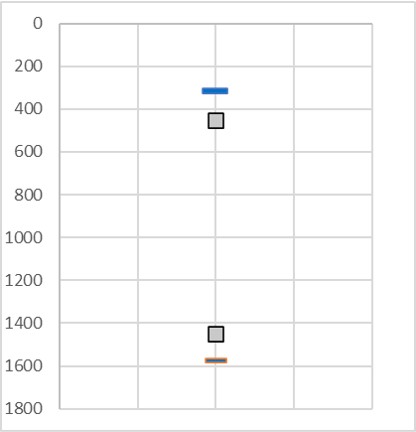

Supplement: Supplementary file 1 [file mmc1.zip › Data_P&A/P_&_A_Plugs/31-5-5.jpg]

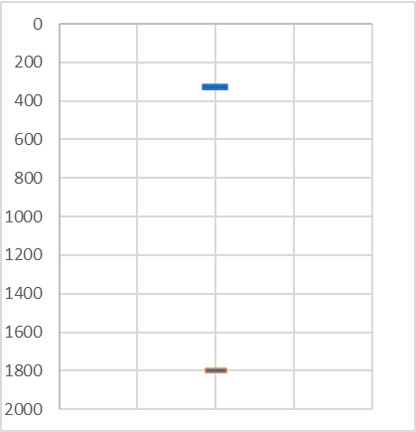

Supplement: Supplementary file 1 [file mmc1.zip › Data_P&A/P_&_A_Plugs/31-5-6.jpg]

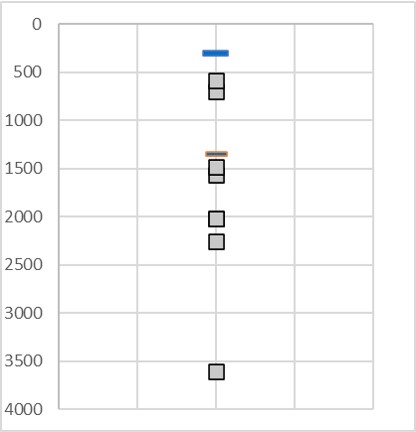

Supplement: Supplementary file 1 [file mmc1.zip › Data_P&A/P_&_A_Plugs/31-6-1.jpg]

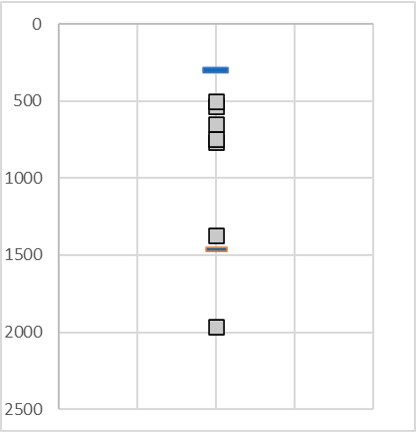

Supplement: Supplementary file 1 [file mmc1.zip › Data_P&A/P_&_A_Plugs/31-6-2.jpg]

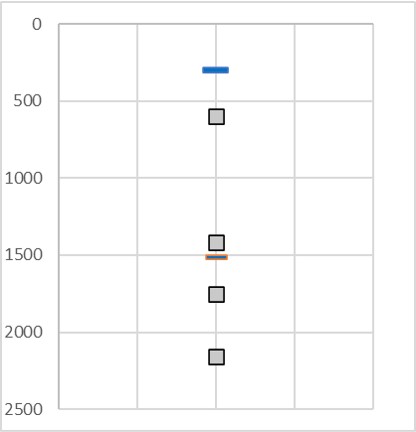

Supplement: Supplementary file 1 [file mmc1.zip › Data_P&A/P_&_A_Plugs/31-6-3.jpg]

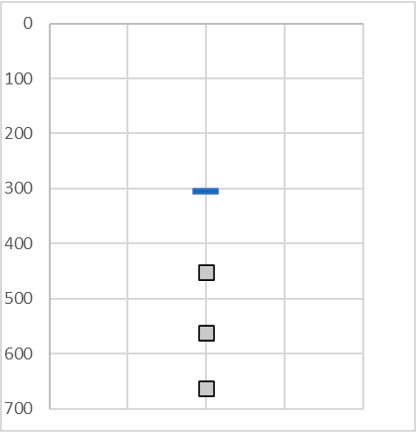

Supplement: Supplementary file 1 [file mmc1.zip › Data_P&A/P_&_A_Plugs/31-6-4.jpg]

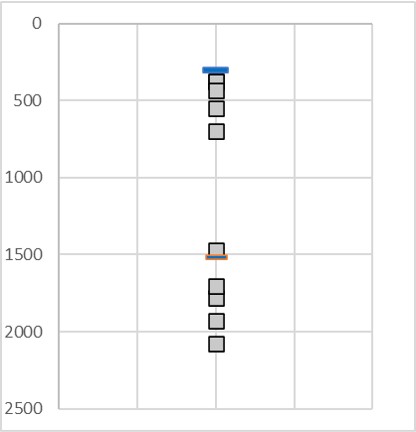

Supplement: Supplementary file 1 [file mmc1.zip › Data_P&A/P_&_A_Plugs/31-6-5.jpg]

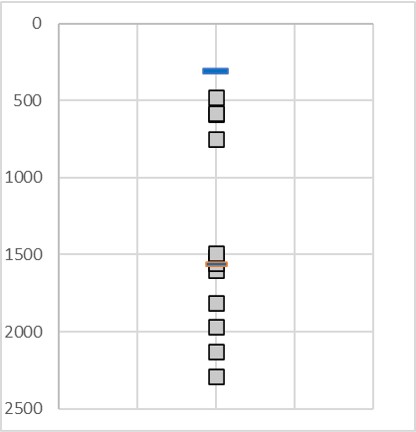

Supplement: Supplementary file 1 [file mmc1.zip › Data_P&A/P_&_A_Plugs/31-6-6.jpg]

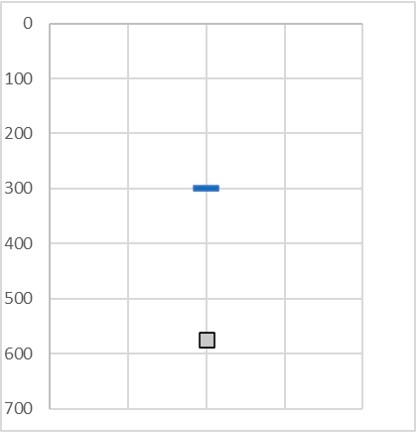

Supplement: Supplementary file 1 [file mmc1.zip › Data_P&A/P_&_A_Plugs/31-6-7.jpg]

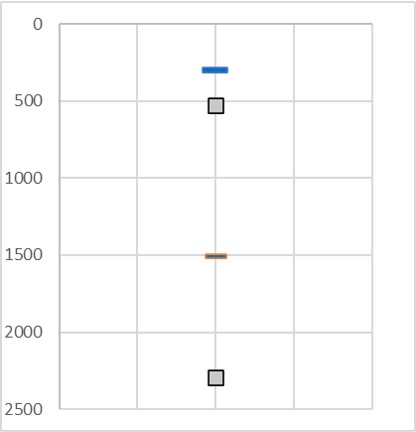

Supplement: Supplementary file 1 [file mmc1.zip › Data_P&A/P_&_A_Plugs/31-6-8.jpg]
